# Molecular structures and in Silico molecular docking of new pyrazine-based heterocycles as antibacterial agents

**DOI:** 10.1186/s13065-025-01535-w

**Published:** 2025-06-11

**Authors:** Mohamed R. Elmorsy, Sara H. Yousef, Ehab Abdel-Latif, Safa A. Badawy

**Affiliations:** Department of Chemistry, Faculty of Science, Mansoura University, Mansoura, 35516 Egypt

**Keywords:** Pyrazine-based heterocycles, Antibacterial activity, Molecular docking, In silico ADME analysis, Structure–activity relationship (SAR)

## Abstract

Compound 2-(2-cyanoacetamido)pyrazine **(3)** serves as a key precursor for synthesizing various new pyrazine-linked heterocycles, including pyridine, thiazole, pyrazole, chromene, and pyrazolotriazine derivatives. Pyrazine-pyridone analogues **5a-d** were obtained by reacting compound **3** with substituted 2-(arylidene)malononitriles **(4a–d).** Substituted pyrazine-thiazoles (8 and 9) were synthesized by condensation with phenyl isothiocyanate, followed by cyclization using ethyl bromoacetate or chloroacetone. Pyrazine-chromenes (14, 15) and pyrazine-naphthoxazines (16, 17) were prepared by reacting salicylaldehyde and naphthol derivatives. Additionally, pyrazine-pyrazolotriazines **19a** and **19b** were formed by coupling diazotized aminopyrazoles (**18a** and **18b**). The structures of the synthesized compounds were confirmed using IR, ^1^HNMR, and ^13^C NMR spectroscopy. Antibacterial activity was evaluated against gram-positive (*S. aureus* and *B. subtilis*) and gram-negative (E. coli and K. pneumoniae) bacteria. Notably, compound 5c exhibited strong activity against *E. coli* (15 mm), and 5d showed potent inhibition against *S. aureus* (18 mm), comparable to the reference antibiotic gentamicin. Molecular docking studies revealed that pyrazine-pyridone derivative 5d displayed the highest binding affinity (S = -7.4519 kcal/mol, RMSD = 1.2498), attributed to two key interactions: one hydrogen-donor and one π-hydrogen bond with the bacterial target (PDB: 4DUH). These in silico findings suggest that **5d** can effectively bind to a critical bacterial enzyme, reinforcing its potential as a promising antibacterial agent. Moreover, the Swiss ADME study provides an in-depth analysis of the drug-like properties and pharmacokinetic attributes of these compounds, further supporting their potential for drug development. Overall, compound **5d** was the most promising candidate for further antibacterial drug design and optimization.

**Supplementary Information:**

The online version contains supplementary material available at 10.1186/s13065-025-01535-w.

## Introduction

**Fig. 1:**
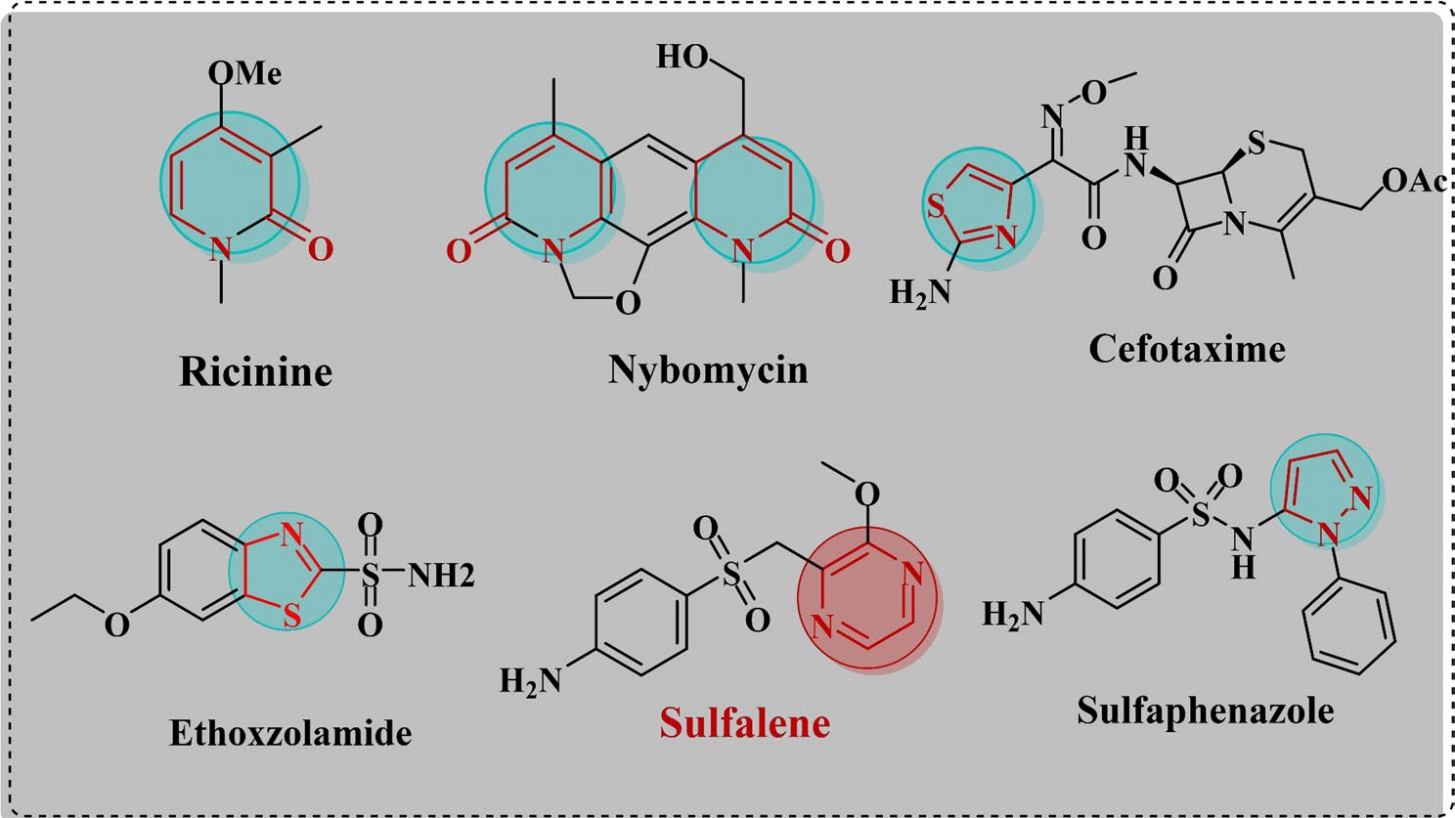
Some antibacterial drugs incorporate 2pyridone, thiazole, pyrazole, and pyrazine rings

Cyanoacetamides are well-known highly active compounds. Their versatile heterocyclic productivity is largely because they include both cyano and carbonyl functional groups, making them excellent reactants and intermediates in a wide variety of reactions. The acidic hydrogen connected to the carbon atom at position 2 also has a significant impact on several condensation and substitution reactions. Biochemists are interested in these chemicals because of their varied biological values and the most approximate way to prepare them [1]. Cyanoacetanilide chemistry has received considerable attention because these reagents are valuable for synthesizing new compounds with various pharmacological properties, such as antibacterial activity, including pyrazole, thiazolidine, oxazine, pyridine, and chromene derivatives [2]. According to reports, heterocyclic compounds made from cyanoacetamides possess beneficial biological characteristics, including analgesic, antifungal, antibacterial, anticancer, and anti-inflammatory effects [3–6]. Nitrogen heterocycles are a significant family of heterocyclic molecules found in many bioactive natural chemicals, indicating their potential use in pharmaceuticals and medical treatments [7, 8]. 2-Pyridones, six-membered nitrogen heterocycles, exhibit diverse biological activities [9]. The anticonvulsant, antibacterial, antioxidant, antiviral, antidiabetic, antimalarial, and anticancer properties of pyridone derivatives have been extensively studied [10–12]. Ricinine and Nybomycin are examples of selective antibacterial agents containing 2-pyridones (Fig. 1) [13, 14]. Thiazole, a sulfur- and nitrogen-containing heterocyclic molecule, has garnered considerable interest because of its extensive biological applications. Thiazole derivatives [15] have been reported to exhibit anti-inflammatory [16], antifungal [17], anticancer [18], antibacterial [19], anticonvulsant [20], antiviral [21], and antioxidant activities [22]. Their versatility makes them valuable tools in the quest for new drugs. The therapeutic potential of thiazole and its derivatives is highlighted by the extensive variety of biological actions associated with them [23]. Cefotaxime and Ethoxzolamide are examples of selective compounds that contain thiazole rings with antibacterial activity (Fig. 1). Cefotaxime is a commercially available third-generation broad-spectrum antibiotic. It is effective against infections caused by both types of bacteria [24]. Pyrazole, possessing the chemical formula C_3_H_4_N_2_, is a distinctive and versatile heterocyclic compound extensively utilized in various biological fields. The medicinal properties of pyrazole derivatives have been extensively investigated [25]. Numerous therapeutic effects have been documented for several pyrazole compounds, including antimicrobial [26], antidiabetic [27], antioxidant [28], anti-inflammatory [29], and anticancer effects [30]. Owing to its extensive therapeutic properties, it has garnered the interest of researchers for further investigation as a potential treatment for numerous diseases [31]. Sulfaphenazole is an example of a pyrazole-containing selective drug with antibacterial activity (Fig. 1) [32]. Oxazines play a significant role as antibacterial, antileishmanial, antiulcer, antitubercular, anticancer, and antihyperglycemic agents [33]. Similarly, pyrazine, another heterocyclic compound, has extensive pharmacological effects [34]. Pyrazine is a promising building block for novel antibacterial medicines. Because of their broad spectrum of biological activities, including antifungal, antibacterial, antidiabetic, diuretic, antiviral, hypnotic, anticancer, and analgesic effects, pyrazine-based compounds are intriguing options for further investigation. The antibacterial activity of pyrazole derivatives has proven to be highly efficient against several types of bacterial and fungal strains [35]. Modifications to pyrazine’s distinctive structural features can result in the development of new, highly effective antibacterial drugs with enhanced pharmacokinetic characteristics [36]. These compounds frequently interfere with essential biological processes, such as cell wall production and protein synthesis, resulting in the death of bacterial and fungal cells. Pharmaceutical compounds derived from pyrazine are promising weapons in the fight against drug-resistant diseases because of their capacity to inhibit the growth of drug-resistant pathogens [35]. Building on the features mentioned earlier and maintaining our emphasis on physiologically effective heterocyclic structures, we introduce new pyrazine hybrids that combine pyridine, triazole, pyrazole, chromene, and pyrazolotriazine moieties using a series of conventional synthetic strategies.

## Experimental

### Synthesis of 2-(2-cyanoacetamido)pyrazine (3)

A mixture of 2-aminopyrazine (**1**) (0.38 g, 4 mmol) and 1-(cyanoacetyl)-3,5-dimethylpyrazole (**2**) (0.65 g, 4 mmol) was boiled in toluene (20 mL) for 4 h. The solid generated during heating was collected to obtain the corresponding pyrazine-cyanoacetamide compound **3** [37].

Brown crystals (65% yield); m.p. = 210–212 °C, lit. m.p. = 191–192 °C [37].

### Synthesis of 6-Amino-4-aryl-2-oxo-1-(pyrazin-2-yl)-1,2-dihydropyridine-3,5-dicarbonitrile derivatives 5a-d

A 50 mL RB-flask was charged with 2-(2-cyanoacetamido)pyrazine **(3)** (0.32 g, 2 mmol) and 2-arylidene-malononitrile compounds **4a-d** (2 mmol) in 30 mL of ethanol containing piperidine (0.1 mL). The solid product generated after refluxing for 2 h was filtered and washed with ethanol to afford the corresponding pyrazine-pyridine compounds **5a-d**.

### 6-Amino-2-oxo-1-(pyrazin-2-yl)-4-(p-tolyl)-1,2-dihydropyridine-3,5-dicarbonitrile (5a)

Buff solid (54% yield); m.p. > 300 °C. IR (*ν*_*max*_.cm^-1^): 3370 (N-H), 2218 (C ≡ N), 1649 (C = O). ^1^H NMR: *δ* 2.40 (s, 3 H, CH_3_), 7.38 (d, *J* = 7.5 Hz, 2 H), 7.45 (d, *J* = 7.5 Hz, 2 H), 8.32 (br. s, 2 H, -NH_2_), 8.79 (s, 1H, pyrazinyl-H), 8.85 (d, *J* = 2.0 Hz, 1H, pyrazinyl-H), 8.90 ppm (s, 1H, pyrazinyl-H). ^13^C NMR: *δ* 20.99, 75.64, 87.60, 115.50, 116.02, 127.92 (2 C), 129.24 (2 C), 131.60, 140.39, 144.52, 145.43, 145.75, 146.38, 156.72, 159.66, 162.30 ppm. Analysis for C_18_H_12_N_6_O (328.11): Calculated: C, 65.85; H, 3.68; N, 25.60%. Found: C, 66.02; H, 3.75; N, 25.72%.

### 6-Amino-4-(4-methoxyphenyl)-2-oxo-1-(pyrazin-2-yl)-1,2-dihydropyridine-3,5-dicarbonitrile (5b)

Gray solid (56% yield); m.p. > 300 °C. IR (*ν*_*max*_.cm^-1^): 3362 (N-H), 2214 (C ≡ N), 1638 (C = O). ^1^H NMR: δ 3.84 (s, 3 H, OCH_3_), 7.13 (d, *J* = 8.5 Hz, 2 H), 7.53 (d, *J* = 9.0 Hz, 2 H), 8.30 (br. s, 2 H, -NH_2_), 8.79 (d, *J* = 1.5 Hz, 1H, pyrazinyl-H), 8.85 (d, *J* = 2.5 Hz, 1H, pyrazinyl-H), 8.89 ppm (s, 1H, pyrazinyl-H). ^13^C NMR: δ 55.38, 75.62, 87.47, 114.05 (2 C), 115.68, 116.20, 126.38, 129.84 (2 C), 144.55, 145.42, 145.76, 146.35, 156.73, 159.74, 160.95, 161.91 ppm. Analysis for C_18_H_12_N_6_O_2_ (344.10): Calculated: C, 62.79; H, 3.51; N, 24.41%. Found: C, 63.01; H, 3.60; N, 24.30%.

### 6-Amino-4-(4-chlorophenyl)-2-oxo-1-(pyrazin-2-yl)-1,2-dihydropyridine-3,5-dicarbonitrile (5c)

Buff solid (52% yield); m.p. > 300 °C. IR (*ν*_*max*_.cm^-1^): 3358 (N-H), 2219 (C ≡ N), 1668 (C = O). ^1^H NMR (DMSO*-d*_*6*_): *δ* 7.60 (d, *J* = 7.0 Hz, 2 H), 7.67 (d, *J* = 7.0 Hz, 2 H), 8.40 (br. s, 2 H, -NH_2_), 8.80 (s, 1H, pyrazinyl-H), 8.86 (s, 1H, pyrazinyl-H), 8.89 ppm (s, 1H, pyrazinyl-H). ^13^C NMR: *δ* 75.69, 87.75, 115.27, 115.78, 128.92 (2 C), 129.94 (2 C), 133.35, 135.35, 144.43, 145.48, 145.69, 146.45, 156.70, 159.50, 161.11 ppm. Analysis for C_17_H_9_ClN_6_O (348.05): Calculated: C, 58.55; H, 2.60; N, 24.10%. Found: C, 58.37; H, 2.67; N, 24.19%.

### 6-Amino-4-(4-nitrophenyl)-2-oxo-1-(pyrazin-2-yl)-1,2-dihydropyridine-3,5-dicarbonitrile

***(5d****)*.

Brown solid (51% yield); m.p. > 300 °C. IR (*ν*_*max*_.cm^-1^): 3358 (N-H), 2219 (C ≡ N), 1669 (C = O). ^1^H NMR: *δ* 7.88 (d, *J* = 8.5 Hz, 2 H), 8.43 (d, *J* = 8.5 Hz, 2 H), 8.81 (s, 1H, pyrazinyl-H), 8.87 (d, *J* = 2.0 Hz, 1H, pyrazinyl-H), 8.90 ppm (s, 1H, pyrazinyl-H). ^13^C NMR: *δ* 75.52, 87.81, 115.99, 115.50, 124.01 (2 C), 129.72 (2 C), 140.76, 144.32, 145.54, 145.63, 146.55, 148.62, 156.72, 159.34, 160.35 ppm. Analysis for C_17_H_9_N_7_O_3_ (359.08): Calculated: C, 56.83; H, 2.52; N, 27.29%. Found: C, 56.98; H, 2.47; N, 27.18%.

### Synthesis of 6-amino-2-oxo-1-(pyrazin-2-yl)-1,2-dihydropyridine-3,5-dicarbonitrile (7)

To a 50 mL RB-flask containing 20 mL of ethanol and sodium metal (0.04 g, 2 mmol), 2-(2-cyanoacetamido)pyrazine (**3**) **(**0.16 g, 1 mmol), and 2-ethoxymethylenemalononitrile (**6**) (0.12 g, 1 mmol) were added. The mixture was then refluxed for 2 h and diluted with 20 mL of cold water. The precipitate formed was subsequently filtered and crystallized from ethanol.

Pale yellow solid (54% yield); m.p. > 300 °C. IR (*ν*_*max*_.cm^-1^): 3366, 3303 (N-H), 2222 (C ≡ N), 1669 (C = O). ^1^H NMR: *δ* 8.40 (s, 1H, =CH), 8.76–8.77 (dd, J = 2.5, 1.5 Hz, 1H, pyrazinyl-H), 8.84 (d, *J* = 3.0 Hz, 1H, pyrazinyl-H), 8.85 ppm (d, *J* = 1.0 Hz, 1H, pyrazinyl-H). ^13^C NMR: *δ* 73.92, 86.83, 115.66, 116.22, 144.42, 145.43, 145.70, 146.40, 151.01, 157.29, 159.65 ppm. Analysis for C_11_H_6_N_6_O (238.06): Calculated: C, 55.46; H, 2.54; N, 35.28%. Found: C, 55.24; H, 2.46; N, 35.14%.

### Synthesis of pyrazine-thiazole hybrids 8 and 9

A mixture of 2-(2-cyanoacetamido)pyrazine (**3**) (0.32 g, 2 mmol) and phenyl isothiocyanate (2 mmol, 0.27 mL) was stirred for 6 h in 20 mL DMF and K_2_CO_3_ (0.54 g, 4 mmol). Then, ethyl bromoacetate (0.34 mL, 2 mmol) or chloroacetone (0.18 mL, 2 mmol) was added, and the mixture was stirred overnight. When the mixture was diluted with 40 mL of cold water, a precipitate was obtained and filtered. The solid was purified by recrystallization from an ethanol solution.

### 2-Cyano-2-(4-oxo-3-phenylthiazolidin-2-ylidene)-N-(pyrazin-2-yl)acetamide (8)

Dark red solid (56% yield); m.p.= 240–242 °C. IR (*ν*_*max*_.cm^− 1^): 3401 (N-H), 2191 (C ≡ N), 1744, 1669 (2 C = O). ^1^H NMR: *δ* 4.04 (s, 2 H, CH_2_), 7.43 (d, *J* = 7.5 Hz, 2 H), 7.52–7.56 (m, 3 H), 8.38 (s, 2 H, pyrazinyl-H), 9.15 (s, 1H, pyrazinyl-H), 9.44 ppm (s, 1H, N-H). ^13^C NMR: *δ* 32.08, 77.88, 113.45, 129.39 (2 C), 129.48 (2 C), 130.67, 134.90, 136.64, 140.26, 142.76, 147.96, 163.28, 171.64, 173.38 ppm. Analysis for C_16_H_11_N_5_O_2_S (337.06): Calculated: C, 56.97; H, 3.29; N, 20.76%. Found: C, 66.17; H, 3.21; N, 20.65%.

### 2-Cyano-2-(4-methyl-3-phenylthiazol-2(3 H)-ylidene)-N-(pyrazin-2-yl)acetamide (9)

Brown crystal solid (72% yield); m.p.= 200–202 °C. IR (*ν*_*max*_.cm^− 1^): 3419 (N-H), 2195 (C ≡ N), 1640 (C = O). ^1^H NMR: *δ* 2.08 (s, 3 H, CH_3_), 7.12 (t, *J* = 7.0 Hz, 1H), 7.35–7.40 (m, 4 H), 7.48 (s, 1H, =CH), 8.33 (s, 1H, pyrazinyl-H), 8.37 (s, 1H, pyrazinyl-H), 9.36 (s, 1H, pyrazinyl-H), 9.81 (s, 1H, N-H). ^13^C NMR: *δ* 28.06, 96.27, 105.16, 120.49, 124.18, 129.41 (3 C), 136.89, 139.35, 140.94, 142.51, 149.05, 154.50, 159.01, 162.94, 186.84 ppm. Analysis for C_17_H_13_N_5_OS (335.08): Calculated: C, 60.88; H, 3.91; N, 20.88%. Found: C, 60.71; H, 3.83; N, 20.75%.

### 2-Cyano-3-(methylthio)-3-(phenylamino)-N-(pyrazin-2-yl)acrylamide (10)

A mixture of 2-(2-cyanoacetamido)pyrazine (**3**) (0.32 g, 2 mmol), phenyl isothiocyanate (2 mmol, 0.27 mL), DMF (20 mL), and KOH (0.12 g, 2 mmol) was stirred for 6 h. Then, iodomethane (0.28 mL, 2 mmol) was added dropwise, and stirring was continued overnight. A precipitate was formed when the mixture was diluted with 40 mL of cold water. The solid was subsequently filtered and crystallized from boiling ethanol.

Orange solid (78% yield); m.p.= 190–192 °C. IR (*ν*_*max*_.cm^− 1^): 3381 (N-H), 2922 (C-H aliphatic), 2191 (C ≡ N), 1652 (C = O). ^1^H NMR: *δ* 2.32 (s, 3 H, CH_3_), 7.14 (t, *J* = 7.5 Hz, 1H), 7.29–7.34 (m, 4 H), 8.30 (d, *J* = 2.5 Hz, 1H, pyrazinyl-H), 8.35 (s, 1H, pyrazinyl-H), 8.96 (s, 1H, pyrazinyl-H), 9.98 (s, 1H, N-H), 11.13 ppm (br. s, 1H, N-H). ^13^C NMR: *δ* 16.45, 79.33, 118.59, 123.17, 125.84, 129.16 (3 C), 136.59, 138.97, 139.59, 142.51, 148.38, 163.88, 168.04 ppm. Analysis for C_15_H_13_N_5_OS (311.08): Calculated: C, 57.86; H, 4.21; N, 22.49%. Found: C, 58.02; H, 4.28; N, 22.39%.

### 5-Amino-3-(phenylamino)-N-(pyrazin-2-yl)-1 H-pyrazole-4-carboxamide (11)

Ketene-N, S-acetal compound **10** (0.31 g, 1 mmol) was boiled with hydrazine hydrate (0.12 mL, 2.5 mmol) in ethanol (20 mL) for 2 h. The precipitate that formed upon cooling was filtered and crystallized by boiling in ethanol to give the conforming aminopyrazole compound **11**.

Buff solid (52% yield); m.p.= 236–238 °C. IR (*ν*_*max*_.cm^− 1^): 3418, 3357, 3325, 3264, 3218 (-NH_2_ and N-H), 1661 (C = O). ^1^H NMR: *δ* 6.19 (br. s, 2 H, exchanged by D_2_O, NH_2_), 6.77 (t, *J* = 6.0 Hz, 1H), 7.13–7.19 (m, 4 H), 8.28–8.32 (m, 3 H, 2 pyrazine-H and N-H exchanged by D_2_O), 9.38 (s, 1H, pyrazine-H), 9.49 (s, 1H, exchanged by D_2_O, N-H), 11.57 ppm (s, 1H, exchanged by D_2_O, N-H). ^13^C NMR: *δ* 88.81, 115.88 (2 C), 119.41, 128.90 (2 C), 136.04, 139.11, 142.61, 144.28, 148.66, 149.25, 150.20, 162.54 ppm. Analysis for C_14_H_13_N_7_O (295.12): Calculated: C, 56.94; H, 4.44; N, 33.20%. Found: C, 57.12; H, 4.34; N, 33.06%.

### Pyrazine-chromene derivatives 14 and 15

2-Hydroxybenzaldehyde **12a** (0.12 g, 1 mmol) and 2-hydroxy-1-naphthaldehyde **12b** (0.17 g, 1 mmol) were added to a suspension of compound **3** (0.16 g, 1 mmol) in 25 mL of ethanol with a drop of piperidine. After 2 h of refluxing the mixture, a precipitate was formed, which was then filtered and washed with ethanol to yield products **14** and **15**.

### 2-Imino-N-(pyrazin-2-yl)-2 H-chromene-3-carboxamide (14)

Pale yellow solid (51% yield); m.p. > 300 °C. IR (*ν*_*max*_.cm^− 1^): 3215 (N-H), 1685 (C = O). ^1^H NMR: *δ* 7.28–7.35 (m, 1H), 7.64 (t, *J* = 8.0 Hz, 1H), 7.80–7.88 (m, 2 H), 8.45 (s, 2 H, pyrazine-H), 8.68 (s, 1H, pyrazine-H), 9.37 (s, 1H, chromene-H4), 9.54 (s, 1H, N-H), 13.49 ppm (s, 1H, N-H). Analysis for C_14_H_10_N_4_O_2_ (266.08): Calculated: C, 63.15; H, 3.79; N, 21.04%. Found: C, 63.01; H, 3.71; N, 21.14%.

### 2-Imino-N-(pyrazin-2-yl)-2 H-benzo[g]chromene-3-carboxamide (15)

Yellow solid (76% yield); m.p. > 300 °C. IR (*ν*_*max*_.cm^− 1^): 3257, 3206 (N-H), 1690 (C = O). ^1^H NMR: *δ* 7.46 (d, *J* = 9.0 Hz, 1H), 7.61 (t, *J* = 8.0 Hz, 1H), 7.76 (t, *J* = 8.0 Hz, 1H), 8.03 (d, *J* = 8.0 Hz, 1H), 8.21 (d, *J* = 8.5 Hz, 1H), 8.42 (s, 2 H, chromene-H4 and pyrazine-H), 8.49 (d, *J* = 9.0 Hz, 1H), 9.18 (s, 1H, pyrazine-H), 9.26 (s, 1H, pyrazine-H), 9.55 (s, 1H, N-H), 13.38 ppm (s, 1H, N-H). Analysis for C_18_H_12_N_4_O_2_ (316.10): Calculated: C, 68.35; H, 3.82; N, 17.71%. Found: C, 68.52; H, 3.88; N, 17.63%.

### Synthesis of pyrazine-naphthoxazine derivatives 16 and 17

2-Nitroso-1-naphthol **13a** (0.17 g, 1 mmol) and 1-nitroso-2-naphthol **13b** (0.17 g, 1 mmol) were added to a suspension of compound **3** (0.16 g, 1 mmol) in ethanol (25 mL) and piperidine (0.1 mL). The mixture was then subjected to reflux for 4 h. The product formed after cooling was collected, dried, and crystallized from ethanol to afford pyrazine-naphthoxazine compounds **16** and **17**, respectively.

### 2-Imino-N-(pyrazin-2-yl)-2 H-naphtho[1,2-b][1, 4]oxazine-3-carboxamide (16)

Blue solid (64% yield); m.p. > 300 °C. IR (*ν*_*max*_.cm^− 1^): 3359, 3307 (N-H), 1622 (C = O). ^1^H NMR: *δ* 7.61–7.66 (m, 2 H), 7.73 (d, *J* = 8.5 Hz, 1H), 7.78–7.86 (m, 3 H), 7.97 (d, *J* = 9.0 Hz, 1H), 8.18 (d, *J* = 6.5 Hz, 1H), 8.63 (d, *J* = 8.0 Hz, 1H), 8.87–8.88 ppm (dd, *J* = 6.5, 3.5 Hz, 1H). Analysis for C_17_H_11_N_5_O_2_ (317.09): Calculated: C, 64.35; H, 3.49; N, 22.07%. Found: C, 64.46; H, 3.43; N, 22.16%.

### 2-Imino-N-(pyrazin-2-yl)-2 H-naphtho[2,3-b][1, 4]oxazine-3-carboxamide (17)

Green solid (66% yield); m.p. > 300 °C. IR (*ν*_*max*_.cm^− 1^): 3351, 3307 (N-H), 1665 (C = O). ^1^H NMR: *δ* 7.49 (t, *J* = 7.5 Hz, 1H), 7.54 (t, *J* = 7.5 Hz, 1H), 7.77 (d, *J* = 9.0 Hz, 1H), 7.83 (d, *J* = 8.5 Hz, 1H), 7.91 (d, *J* = 8.0 Hz, 1H), 8.65–8.68 (m, 2 H, Ar-H, pyrazinyl-H), 8.79 (s, 1H, pyrazinyl-H), 8.97 ppm (s, 1H, pyrazinyl-H). Analysis for C_17_H_11_N_5_O_2_ (317.09): Calculated: C, 64.35; H, 3.49; N, 22.07%. Found: C, 64.20; H, 3.57; N, 22.20%.

### Synthesis of 4-Imino-7-substituted-N-(pyrazin-2-yl)-1,4-dihydropyrazolo[5,1-c][1, 2, 4]triazine-3-carboxamide derivatives 19a and 19b

A solution of NaNO_2_ (0.14 g in 5 mL H_2_O) was dropped gradually to a cold suspension (0–5 °C) of aminopyrazole compound; either 3-methyl-1*H*-pyrazol-5-amine (**18a**) (2 mmol, 0.19 g) or 3-phenyl-1*H*-pyrazol-5-amine (**18b**) (2 mmol, 0.32 g) in conc. HCl (0.6 mL). The synthesized diazonium solution was then added to a suspension of pyrazinyl cyanoacetamide **3** in 25 mL of ethanol and sodium acetate (0.60 g). The mixture was maintained at 0–5 °C for 2 h. The solid obtained after filtration was crystallized from ethanol (20 mL) and acetic acid (1 mL) to furnish the desired pyrazolotriazine compounds **19a** and **19b**.

### 4-Amino-7-methyl-N-(pyrazin-2-yl)pyrazolo[5,1-c][1, 2, 4]triazine-3-carboxamide (19a)

Yellowish-brown crystals (72% yield); m.p. = 280–282 °C. IR (*ν*_*max*_.cm^-1^): 3318, 3264 (N-H), 1671 (C = O). ^1^H NMR: *δ* 2.50 (s, 3 H, CH_3_), 6.92 (s, 1H, pyrazole-H), 8.43 (d, *J* = 3.0 Hz, 1H, pyrazinyl-H), 8.47 (t, *J* = 2.00 Hz, 1H, pyrazinyl-H), 8.87 (br. s, 1H), 9.32 (br. s, 1H) (NH_2_), 9.41 ppm (s, 1H, pyrazinyl-H), 10.57 (s, 1H, N-H). ^13^C NMR: *δ* 14.31, 97.95, 117.56, 136.50, 140.29, 141.12, 142.99, 147.65, 149.85, 156.62, 164.34 ppm. Analysis for C_11_H_10_N_8_O (270.10): Calculated: C, 48.89; H, 3.73; N, 41.46%. Found: C, 48.70; H, 3.64; N, 41.60%.

### 4-Amino-7-phenyl-N-(pyrazin-2-yl)pyrazolo[5,1-c][1, 2, 4]triazine-3-carboxamide (19b)

Orange solid (78% yield); m.p. > 300 °C. IR (*ν*_*max*_.cm^-1^): 3373, 3323, 3271 (N-H), 1665 (C = O). ^1^H NMR: *δ* 7.13 (s, 1H, pyrazole-H), 7.47–7.51 (m, 3 H), 7.85–7.87 (m, 2 H), 8.45 (d, *J* = 2.5 Hz, 1H, pyrazinyl-H), 8.50 (t, *J* = 2.5 Hz, 1H, pyrazinyl-H), 9.02 (br. s, 1H), 9.33 (br. s, 1H) (-NH_2_), 9.44 (s,1H, pyrazinyl-H), 10.65 ppm (s, 1H, NH). Analysis for C_16_H_12_N_8_O (332.11): Calculated: C, 57.83; H, 3.64; N, 33.72%. Found: C, 57.66; H, 3.67; N, 33.83%.

### Biological evaluation

#### Antibacterial evaluation

The antibacterial efficacy of the targeting pyrazine-based hybrids was evaluated against diverse pathogenic bacteria, including Gram + ve bacteria *S. aureus* and *B. subtilis*, Gram -ve bacteria *K. pneumonia*, and *E. coli*. Bacterial strains were inoculated onto agar plates and assessed for susceptibility using the well diffusion method. A total of 100 µL of each sample was introduced into each well and incubated for 24–48 h at 37 °C. DMSO (negative control) was used to dissolve the tested substances [38].

#### Molecular Docking study

Molecular docking is an auxiliary tool for understanding the interactions between a ligand and the critical active regions of a protein [39]. Docking simulations and associated computational studies were performed using the Molecular Operating Environment (MOE) 2019 software. The crystal structure of *Escherichia coli* DNA gyrase subunit B (PDB ID: 4DUH), representing the 24 kDa domain of the enzyme, was used as the docking model because of its critical role in bacterial DNA replication and transcription. DNA gyrase is an essential bacterial enzyme responsible for introducing negative supercoils into DNA, a process vital for DNA replication and transcription. Inhibition of DNA gyrase disrupts these fundamental bacterial processes, effectively halting bacterial growth and leading to cell death. This makes DNA gyrase a highly specific and validated target for developing antibacterial agents [26]. The docking workflow involved several steps: (1) removing water molecules and non-essential atoms from the target protein; (2) preparing the protein by adding missing hydrogen atoms, automatically assigning atom types, and optimizing the potential energy; and (3) running the docking simulation. Docking was performed within a 10 Å cubic grid along the x, y, and z axes, centered on the ligand, using MOE software. The binding scores for the various compounds were ranked in the following order: 5d > 19b > 5b > 16 > 15 > 5a > 5c > 9 > 17 > 8 > 11 > 19a > 14 > 7. Finally, the binding energies of the ligand-receptor interactions were collected.

#### Swiss ADME study

Many considerations are made regarding the pharmacodynamics of newly reared small molecules during the drug discovery process. However, the pharmacokinetic behavior of a drug candidate is the main focus of its promotion. The synthesized compounds were subjected to in silico ADME screening to determine their probable absorption, distribution, metabolism, and excretion properties [40]. The Swiss Institute of Bioinformatics (SIB)-provided the SwissADME webtool (https://www.swissadme.ch) was used to process the predictions for compounds **(5a–5d**, **7**, **8**, **9**, **11**, **14–17**, **19a**, and **19b**). The key factors assessed were molecular mass (M.M.), hydrogen bond donors (HBD), partition coefficient (LogP), hydrogen bond acceptors (HBA), breaches of Lipinski’s Rule of Five, solubility, gastrointestinal (GI) uptake, bioavailability index, and topological polar surface region (TPSA). Overall, all evaluated molecules demonstrated favorable physicochemical and pharmacokinetic attributes without deviations from Lipinski’s Rule of Five.

## Results and discussion

### Synthesis of pyrazine-based hybrids

**Scheme 1:**
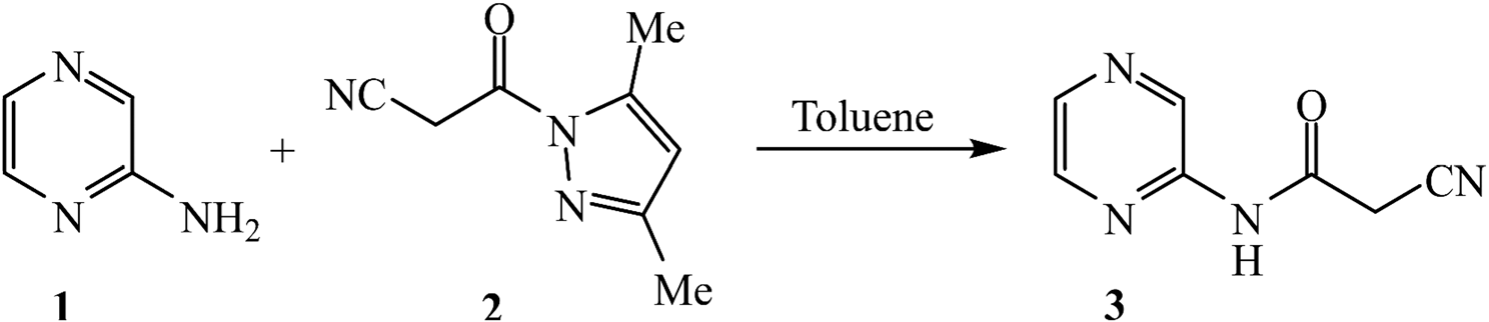
Preparation of 2-(2-cyanoacetamido)pyrazine (**3**)

The key of this study, 2-(2-cyanoacetamido)pyrazine (**3**) [37] was obtained by treatment 2-amniopyrazine (**1**) with 1-cyanoacetyl-3,5-dimethylpyrazole (**2**) in refluxing toluene as indicated in Scheme 1.

**Scheme 2:**
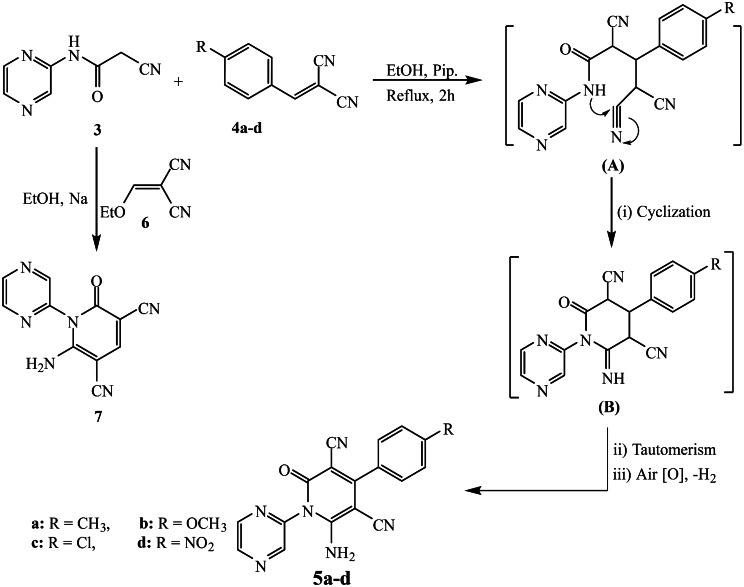
Preparation of pyrazine-pyridine hybrids **5a-d** and **7**

The pyrazinyl-cyanoacetamide compound **3** was refluxed with arylidene malononitriles **4a-d** in ethanol and piperidine to furnish the corresponding pyrazine-pyridine hybrids **5a-d** (Scheme 2). The mechanism was initiated by the Michael addition reaction through the formation of the first intermediate (**A**), followed by nucleophilic addition of the N-H group of the cyanacetamide part into the nitrile group, resulting in cyclization and formation of the second intermediate (B). Finally, intermediate (**B**) undergoes tautomerism and air oxidation to yield the final targeted product, pyrazine-pyridine hybrids **5a-d**. In addition, the addition of 2-(2-cyanoacetamido)pyrazine (**3**) to 2-ethoxymethylenemalononitrile (**6**) resulted in the formation of pyrazine-pyridine hybrid of the type **7** in good yield. The spectroscopic data of the obtained pyrazine-pyridine hybrids **5a-d** and **7** confirmed their assigned structures. For pyrazine-pyridine derivative **5a** (as an example), the infrared spectrum showed the absorption frequencies of the (N-H), nitrile (C ≡ N), and carbonyl (C = O) groups at 3370, 2218, and 1649 cm^− 1^, respectively. Its ^1^H NMR spectrum exhibited the characteristic signals of the new formed pyridine ring as a singlet signal at δ 2.40 ppm corresponding to the protons of the methyl group and doublet signals at δ 7.38 and 7.45 ppm for the phenylene protons. Additionally, a broad signal at δ 8.32 ppm was attributed to the protons of the amino (-NH_2_) group. Moreover, the ^13^C NMR spectrum revealed characteristic signals at δ 20.99 and 162.30 ppm, which correspond to the carbon atoms of the methyl and carbonyl groups, respectively.

**Scheme 3:**
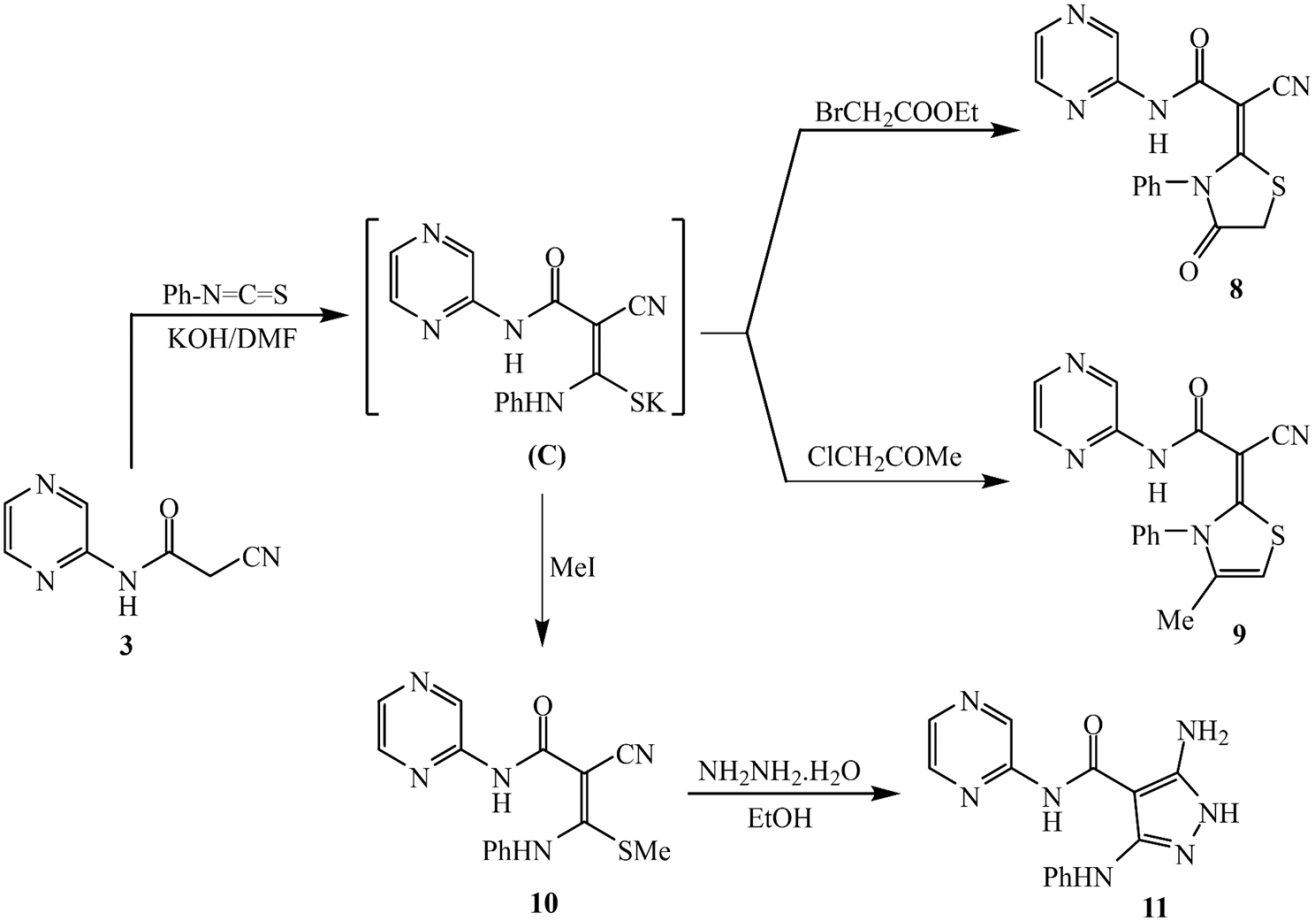
Synthesis of pyrazine hybridized with thiazole or pyrazole derivatives **8**, **9** and **11**

The addition of phenyl isothiocyanate to 2-(2-cyanoacetamido)pyrazine **3** was achieved by stirring in DMF and KOH, leading to the formation of a non-isolated sulfide salt (Intermediate **C**) **(**Scheme 3). This salt undergoes substitution reactions with various α-halogenated reagents (ethyl bromoacetate and chloroacetone), followed by in situ cyclization to form thiazolidin-4-one **8** and thiazoline derivative **9**. The molecular framework of thiazolidine-4-one analog **(8**) was confirmed through ¹H NMR spectrum revealed a characteristic singlet peak corresponding to the methylene (CH₂) hydrogen atoms at δ 4.04 ppm. The protons in the aromatic system were detected as doublet and multiplet signals in the range of δ 7.43–7.56 ppm, while the three protons of the pyrazine ring were identified as two singlet signals at δ 8.38 and 9.15 ppm. In addition, a singlet signal for the N-H proton was detected at δ 9.44 ppm. Furthermore, in situ alkylation of non-isolated potassium sulphide salt **(C)** with iodomethane furnished the corresponding ketene-N, S-acetal compound **10**. Refluxing **10** with hydrazine hydrate in refluxing ethanol provided the corresponding pyrazine-pyrazole (**11)** with a yield of 52%. The IR spectrum of compound **10** indicates the presence of N–H stretching at 3381 cm^− 1^, nitrile (C ≡ N) at 2192 cm^− 1^, and amidic carbonyl at 1652 cm^− 1^. The ^1^H NMR spectrum revealed the protons S-methyl group as a singlet at δ 2.32 ppm. The phenyl protons were detected as a triplet and multiplet signals in the region from 7.14 to 7.34 ppm. The IR spectrum of compound **11** indicated the disappearance of the nitrile group absorption and the presence of -NH_2_ and N-H groups at 3418, 3357, 3325, 3264, and 3218 cm^− 1^. The ^1^H NMR spectrum of also showed the disappearance of the S-methyl protons and exhibited distinctive peaks for amino groups that were exchangeable with D_2_O at δ 6.19 ppm. Furthermore, two protons of the pyrazine ring were observed in the multiplet signal in the range of δ 8.28–8.32 ppm with a proton of the N-H group exchangeable with D_2_O, while the third proton of the pyrazine ring appeared as a singlet signal at δ 9.38 ppm.

**Scheme 4:**
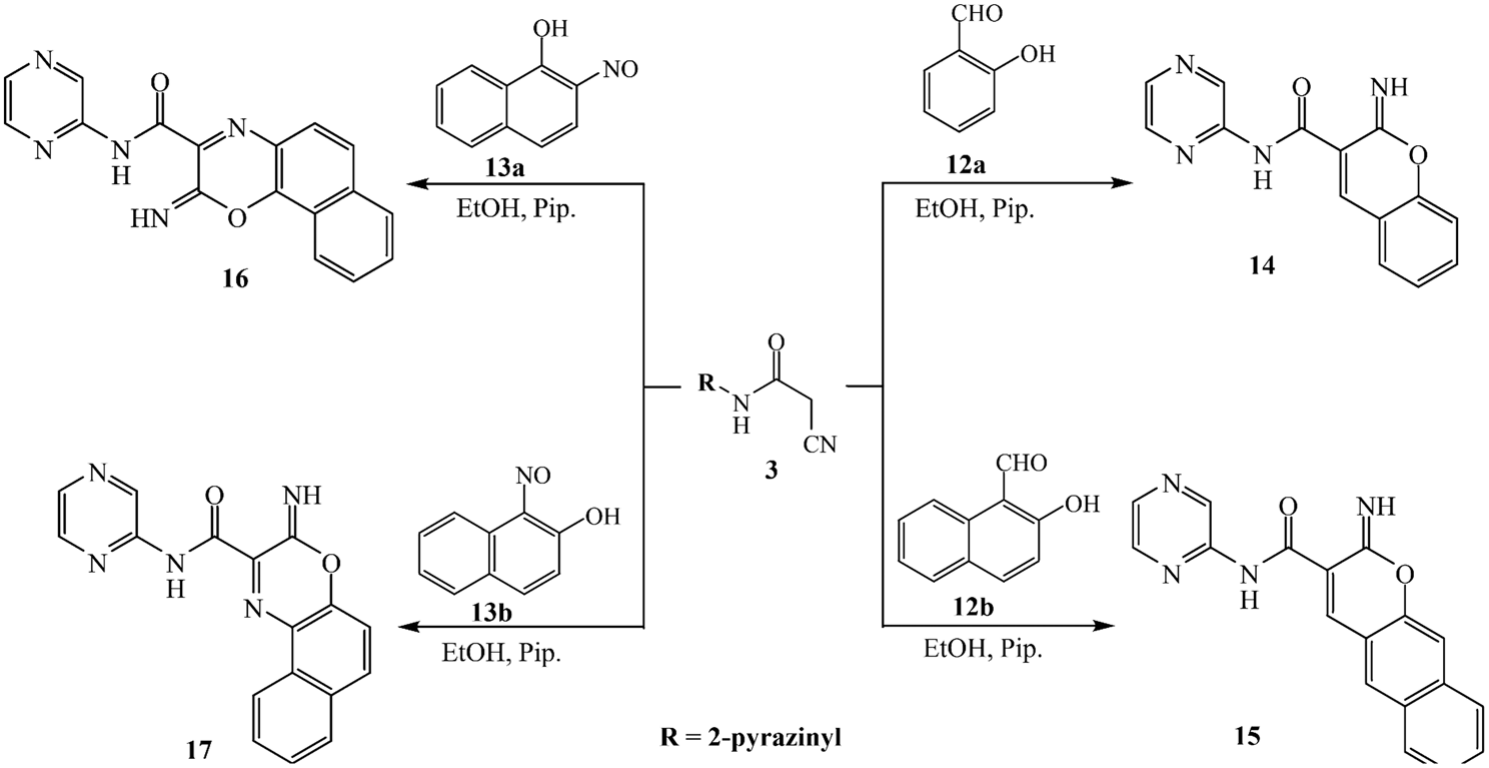
Synthesis of pyrazine-chromene hybrids **14–15** and pyrazine-oxazine hybrids **16–17**

The reaction of 2-(2-cyanoacetamido)pyrazine (**3**) with salicylaldehyde and 2-hydroxy-1-naphthaldehyde proceeded in ethanol and a few drops of piperidine to form the corresponding pyrazine-chromene derivatives **14** and **15** in excellent yields, respectively. The spectroscopic data of the obtained pyrazine-chromene derivatives **14** and **15** confirmed the assigned structures. For pyrazine-chromene derivative **14**, the infrared spectrum indicated the lack of any absorption related to the nitrile group, near 2200 cm^− 1^. The ^1^H NMR spectrum indicates the presence of a singlet signal at 9.37 ppm associated with the olefinic proton of the chromene ring and singlet signals at 9.54 and 13.49 ppm, which correspond to the two protons of the imine N-H and amide N-H, respectively. Furthermore, pyrazine-naphthoxazines **16** and **17** were produced by the cyclocondensation of cyanoacetamido-pyrazine compound **3** with 2-nitroso-1-naphthol **13a** or 1-nitroso-2- naphthol **13b** in boiling ethanol and piperidine, as shown in Scheme 4. The IR spectrum of compound **16** indicated the absence of the nitrile group, while absorption bands appeared at 1622 cm^− 1^, corresponding to the amidic carbonyl group. Moreover, the ^1^H NMR spectrum showed a multiplet signal in the range of δ 7.78–7.86 ppm for three protons of the pyrazine ring.

**Scheme 5:**
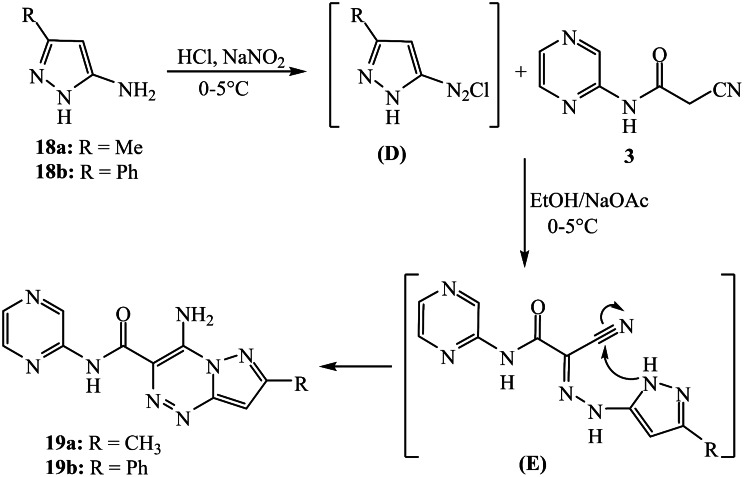
Synthesis of pyrazine-pyrazolotriazine hybrids **19a** and **19b**

As 2-(2-cyanoacetamido)pyrazine (**3**) exhibits a strong tendency for in situ cyclization when coupled with diazotized heterocyclic amines, such as 3-methyl-1*H*-pyrazol-5-amine **18a** and 3-phenyl-1*H*-pyrazol-5-amine **18b**, pyrazine-pyrazolotriazine derivatives **19a** and **19b** are produced in good yields (72 and 78%, respectively) as indicated in Scheme 5. The reaction proceeds by diazotization of aminopyrazole compounds **18a-b** (treatment with nitrous acid) followed by diazocoupling of the produced diazonium salts (intermediate **D**) with cyanoacetamide-pyrazine compound 3 in ethanol and sodium acetate. The intermediate (**E**) undergoes in situ cyclization by nucleophilic addition of N-H (pyrazole) to the nitrile group to roduce the pyrazoltriazine compounds **19a-b**. Analyzing the spectra of the produced pyrazolotriazines **19a** and **19b** confirmed their structures. For the pyrazolotriazine compound **19a** (as an example), the infrared spectrum confirmed that there was no peak for the nitrile group. The ^1^H NMR spectrum demonstrated singlet signals at δ 2.50 ppm for protons of methyl group and for the pyrazole-proton at δ 6.92 ppm. The two singlet signals at δ 8.87 and 9.32 ppm are characteristic for the -NH_2_ protons. The ^13^C NMR spectra displayed distinct signal at δ 164.34 ppm specific to the amidic carbonyl group.

### Antimicrobial study

Based on Table 1, all the newly created derivatives were tested for their effectiveness against the bacteria strains *S. aureus*, *B. subtilis*, *E. coli*, and *K. pneumonia*. The diameter of the zone of inhibition measured in millimeters indicates the antibacterial efficacy. Gentamycin was utilized as the standard drug of reference. Compound **5a** showed minimal antibacterial activity, with only weak effects on *S. aureus* (6 mm) and *B. subtilis* (7 mm), and no impact on *E. coli*, but some inhibition of *K. pneumoniae* (9 mm). This indicates a main emphasis on Gram-positive bacteria, with some efficacy against specific Gram-negative varieties. On the flip side, compound **5b** exhibited minimal antibacterial efficacy, only causing slight inhibition zones of 4 mm for *S. aureus* and 5 mm for *B. subtilis*, with no impact on *E. coli* or *K. pneumoniae*, suggesting a limited range of antibacterial activity. On the other hand, compound **5c** showed specific antibacterial effects, being ineffective against *S. aureus* and *B. subtilis* but showing strong inhibition of *E. coli* (15 mm) and limited activity against *K. pneumoniae* (7 mm), highlighting its effectiveness against *E. coli* and preference for Gram-negative bacteria. Compound **5d** stood out as the top performer in this group, displaying potent antibacterial properties with substantial suppression of *S. aureus* (18 mm), moderate impact on *B. subtilis* (6 mm), and notable effectiveness against both *E. coli* (13 mm) and *K. pneumoniae* (14 mm), demonstrating its wide-ranging capabilities. Likewise, compound **7** showed moderate efficacy against S. aureus (12 mm), low inhibition of both *B. subtilis* (7 mm) and *E. coli* (7 mm), and no impact on *K. pneumoniae*, suggesting moderate effectiveness against Gram-positive bacteria, especially *S. aureus*, with minimal activity against Gram-negative bacteria. Furthermore, compound **8** exhibited distinct antibacterial properties towards *B. subtilis* (12 mm) but did not show any activity against *S. aureus*, *E. coli*, and *K. pneumoniae*. However, compound **9** showed low effectiveness against *S. aureus* (7 mm) but had moderate impact on *B. subtilis* (8 mm), *E. coli* (12 mm), and *K. pneumoniae* (14 mm), demonstrating broad-spectrum efficacy with a particular enhancement against Gram-negative bacteria. Compound **11** displayed low effectiveness against *S. aureus* (6 mm) and moderate hindrance of *E. coli* (11 mm) and *K. pneumoniae* (8 mm), with no impact on *B. subtilis*. This shows a favoritism for Gram-negative bacteria and a decreased efficiency towards Gram-positive strains. On the other hand, compound **14** showed low to medium effectiveness against all bacteria, with respective inhibition zones of 9 mm for *S. aureus*, 10 mm for *B. subtilis*, 8 mm for *E. coli*, and 7 mm for *K. pneumoniae*, indicating a slightly superior effect on Gram-positive bacteria. Compound **15** showed uniform yet feeble efficacy against all strains, displaying inhibition zones measuring 7 mm (*S. aureus*), 8 mm (*B. subtilis*), 8 mm (*E. coli*), and 8 mm (*K. pneumoniae*), suggesting a non-targeted antibacterial mechanism with restricted effectiveness. However, compound **16** exhibited mild effectiveness against various bacterial strains, displaying inhibition zones of 8 mm (*S. aureus*), 9 mm (*B. subtilis*), 9 mm (*E. coli*), and 9 mm (*K. pneumoniae*), indicating potential for a wide range of bacteria but requiring additional refinement for better results. On the other hand, compound **17** showed no activity against *S. aureus*, *B. subtilis*, and *E. coli*, but had slight inhibitory effects on *K. pneumoniae* (10 mm), showing specificity towards *K. pneumoniae* with a limited antibacterial spectrum. Moreover, compound **19a** displayed moderate efficacy against *B. subtilis* (11 mm) and *K. pneumoniae* (12 mm), and weak to moderate efficacy against *S. aureus* (8 mm) and *E. coli* (9 mm), indicating a wide range of activity especially targeting Gram-negative bacteria. On the other hand, compound **19b** demonstrated no effectiveness against the tested bacterial strains, implying minimal antibacterial characteristics and implying that its structure may not be ideal for such uses or may need extensive changes. In conclusion, Gentamycin, acting as the standard antibiotic, showed the largest zones of inhibition against all bacterial strains (19–20 mm), highlighting its superior effectiveness compared to the created compounds.

**Table 1:** Diameter of Inhibition zones(mm)of the synthesized compounds **5a-19b**

Compounds	Gram(+)bacteria		Gram(−)bacteria	
	S. aureus	B. subtilis	E. coli	K. Pneumonia
5a	6	7	N. A.	9
5b	4	5	N. A.	N. A.
**5c**	N. A.	N. A.	**15**	7
**5d**	**18**	6	13	**14**
7	12	7	7	N. A.
8	N. A.	12	N. A.	N. A.
**9**	7	8	12	**14**
11	6	N. A.	11	8
14	9	10	8	7
15	7	8	8	8
16	8	9	9	9
17	N. A.	N. A.	N. A.	10
19a	8	11	9	12
19b	N. A.	N. A.	N. A.	N. A.
**Gentamycin**	19	19	20	20

Where, Gentamycin is the reference; N. A.: No inhibition

### Molecular Docking study

**Fig. 2:**
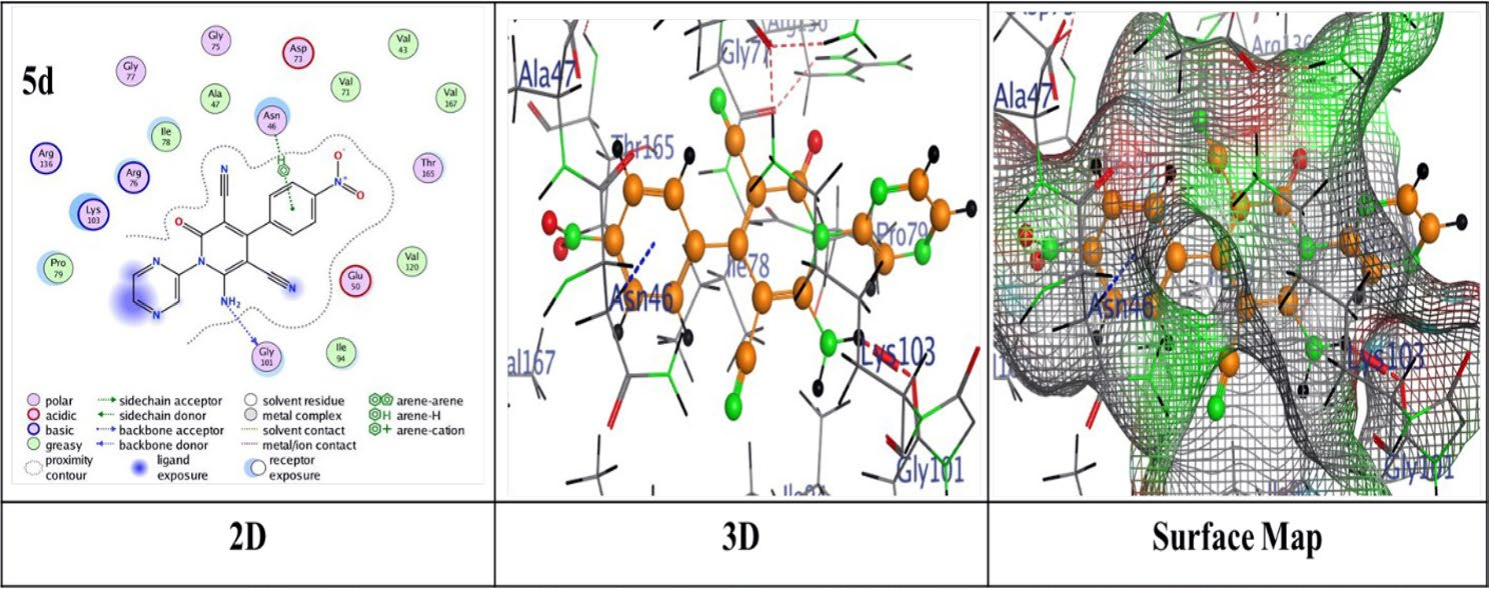
Binding interactions of molecule **5d** with the active regions of (PDB ID: 4DUH)

**Fig. 3:**
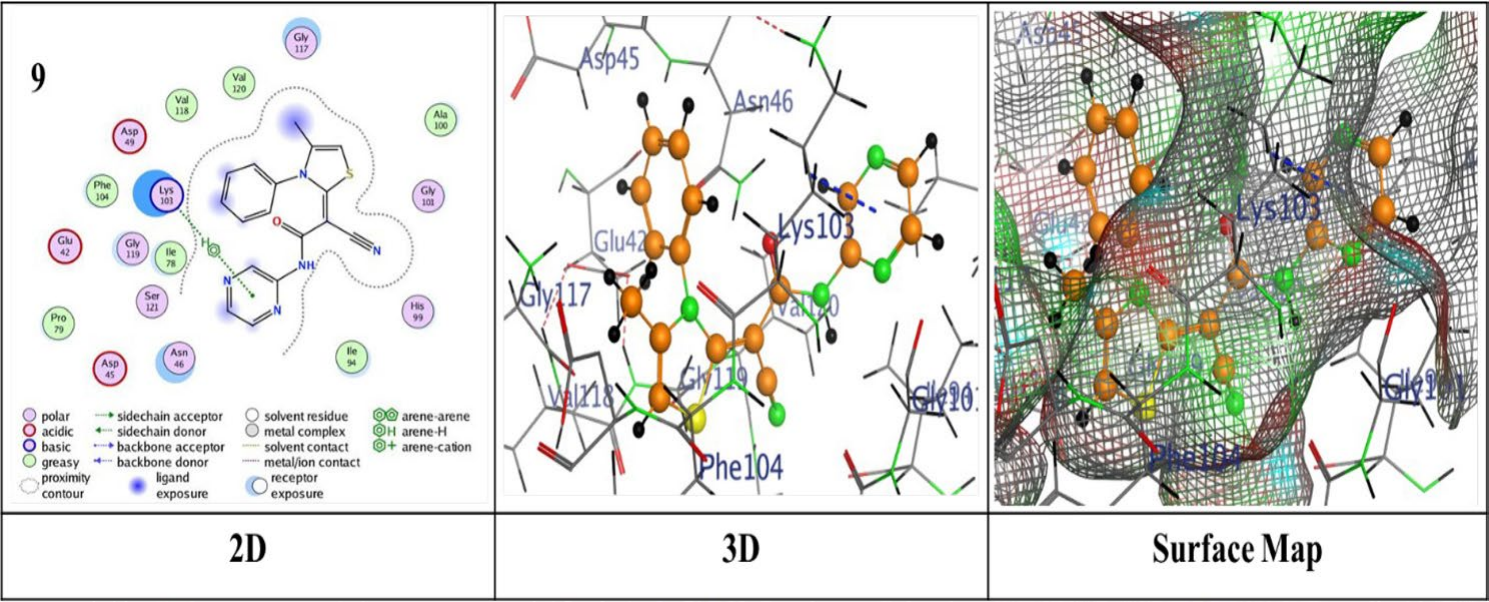
Binding interactions of molecule **9** with the active regions of (PDB ID: 4DUH)

**Fig. 4:**
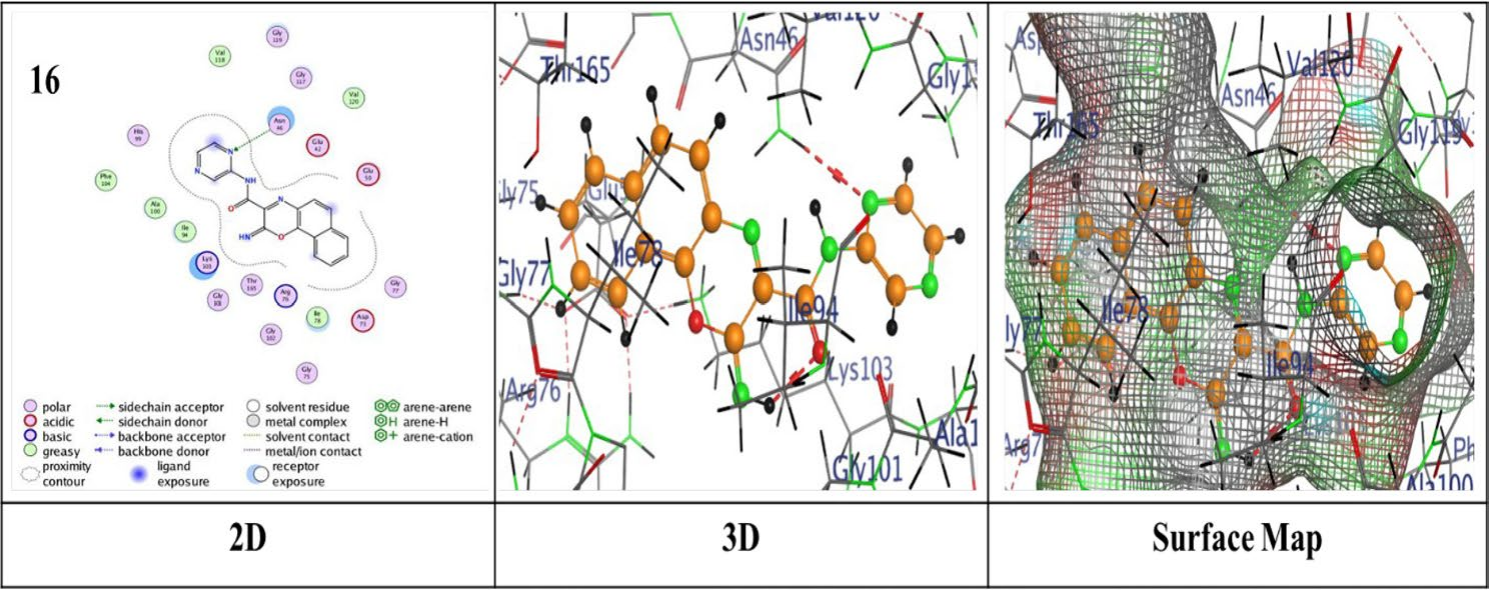
Binding interactions of molecule **16** with the active regions of (PDB ID: 4DUH)

**Fig. 5:**
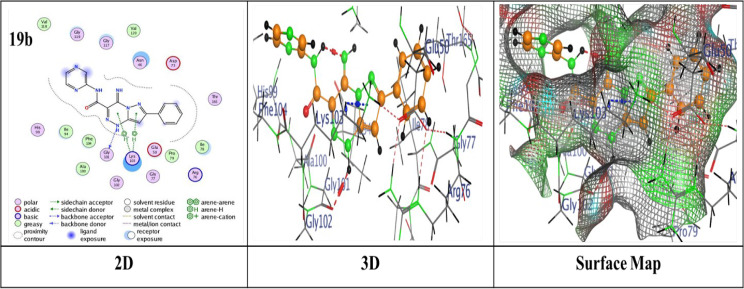
Binding interactions of molecule **19b** with the active regions of (PDB ID: 4DUH)

Thermodynamics in biological systems can be better understood by using molecular docking techniques to assess surface and chemical properties. The bond lengths, docking scores, and ligand interactions with specific amino acids of the target protein (PDB: 4DUH) are presented in Table 2. Typically, docking scores below- 6.0 kcal/mol are regarded as indicative of strong binding interactions, suggesting potential biological activity of the compounds where more negative values suggest stronger and more favorable interactions. From the compiled results, it was observed that the compounds under examination demonstrated an excellent fit within the protein’s active site, yielding binding energy scores ranging from -6.2950 to ‐7.4519 kcal/mol. Pyridine-based analogues (**5a**–**5d**, **7**) exhibited consistent binding via hydrogen and π-hydrogen interactions. Pyridine derivative **5d** demonstrated the highest binding score of -7.4519 kcal/mol, attributed to a hydrogen-donor bond with Gly 101 and a π-hydrogen bond with Asn 46, outperformed the other analogues regarding binding strength (Fig. 2). In comparison, **5b** displayed a binding score of -7.3835 kcal/mol, with two hydrogen-acceptor bonds to Lys 103 and Gly 77 (RMSD = 1.1571) (Additional file 1: Fig. S2). Pyridine analogues **5a** and **5c** showed similar interactions with binding scores of -7.3018 and − 7.2467 kcal/mol, respectively, involving hydrogen-donor bonds with Asp 73 and π-hydrogen bonds with Asn 46 (Additional file 1: Figs. S1 and S3). On the other hand, Pyridine analogue **7** achieved the lowest binding score of -6.2950 kcal/mol (Additional file 1: Fig. S4). Thiazole-based analogues **8** and **9** showed notable binding interactions; compound **8** showed a score of -6.9222 kcal/mol through hydrogen-donor and acceptor interactions with Gly 117 and Ser 121 (Additional file 1: Fig. S5), while compound **9** achieved a better score of -7.1973 kcal/mol and an RMSD of 1.3146, attributed to a π-H bond with Lys 103 (Fig. 3). Pyrazole derivative **11** exhibited a reasonable binding profile (S = ‐6.9026 kcal/mol, RMSD = 1.3146) through a hydrogen-donor interaction with Gly 101 (Additional file 1: Fig. S6). Analogue **14** showed weaker affinity (S = ‐6.7530 kcal/mol, RMSD = 1.8810), driven by a single hydrogen-acceptor bond with Lys (Additional file 1: Fig. S7). In contrast, compound **15** achieved a more favorable score (S = ‐7.3325 kcal/mol, RMSD = 1.0317), owing to a hydrogen-acceptor bond with Val 120 (Additional file 1: Fig. S8). Oxazine analogues **16** and **17** demonstrated solid binding performance (S = ‐7.3501 and ‐7.0912 kcal/mol; RMSD = 1.3130 and 1.3343), with **16** forming a hydrogen-acceptor interaction with Asn 46 (Fig. 4), and **17** engaging in a π-H bond with Ile 78 (Additional file 1: Fig. S9). Triazine derivative **19a** formed four π-H interactions and scored ‐6.7903 kcal/mol (Additional file 1: Fig. S10), while **19b** showed enhanced binding (S = ‐7.4427 kcal/mol), attributed to a hydrogen-donor bond with Gly 101 and two π-H interactions with Lys 103 as illustrated in (Fig. 5).

**Table 2:** Potential Docking results and specific binding of the ligands and protein

Cpd. No.	Binding energy (S) Kcal/mol	RMSD	Distance (Å)	Binding interactions		
				Ligand	Receptor	Interaction type
5a	-7.3018	0.6638	2.90	N20-atom of Amino group	Asp 73	H-donor
			4.64	Pyrid-2-one-ring	Asn 46	π-H
5b	-7.3835	1.1571	3.23	N6-atom of pyrazine ring	Lys 103	H-acceptor
			3.00	N24- nitrile group	Gly 77	H-acceptor
5c	-7.2467	1.5719	2.91	N21-atom of Amino group	Asp 73	H-donor
			4.64	Pyrid-2-one-ring	Asn 46	π-H
5d	-7.4519	1.2498	2.95	N20-atom of Amino group	Gly 101	H-donor
			3.66	Benzene ring	Asn 46	π-H
7	-6.2950	0.7103	3.38	C10-atom of pyridine ring	Asp 73	H-donor
			3.11	N6-atom of pyrazine ring	Asn 46	H-acceptor
			2.99	N16- nitrile group	Gly 77	H-acceptor
			3.94	Pyrazine-ring	Lys 103	π-H
8	-6.9222	1.1637	3.18	C14-atom of thiazole ring	Gly 117	H-donor
			3.67	N18- nitrile group	Ser 121	H-acceptor
9	-7.1973	1.1221	3.88	Pyrazine-ring	Lys 103	π-H
11	-6.9026	1.3146	2.99	N12-atom of pyrazole-ring	Gly 101	H-donor
14	-6.7530	1.8810	3.48	N6-atom of pyrazine ring	Lys 103	H-acceptor
15	-7.3325	1.0317	3.40	O11-atom of carbonyl group	Val 120	H-acceptor
16	-7.3501	1.3130	3.58	N3-atom of pyrazine ring	Asn 46	H-acceptor
17	-7.0912	1.3343	4.21	Benzene ring	Ile 78	π-H
19a	-6.7903	1.3217	3.99	Pyrazole-ring	Asn 46	π-H
			3.71	Triazine ring	Asn 46	π-H
			3.73	Triazine ring	Ile 78	π-H
			3.62	Pyrazine ring	Pro 79	π-H
19b	-7.4427	1.2292	2.99	N11-atom of triazine ring	Gly 101	H-donor
			4.10	Pyrazole-ring	Lys 103	π-H
			3.75	Triazine ring	Lys 103	π-H
Gentamycin	-7.9238	1.7385	3.45	O25-atom of hydroxyl group	Glu 42	H-donor
			3.15	N26-atom of Amino group	Gly 117	H-donor
			3.31	O5-atom of ring	Gly 117	H-acceptor
			3.20	N23-atom of Amino group	Gly 117	H-acceptor
			2.83	O25-atom of hydroxyl group	Asn 46	H-acceptor
			3.04	O33-atom	Asn 46	H-acceptor

### Swiss ADME study

**Fig. 6:**
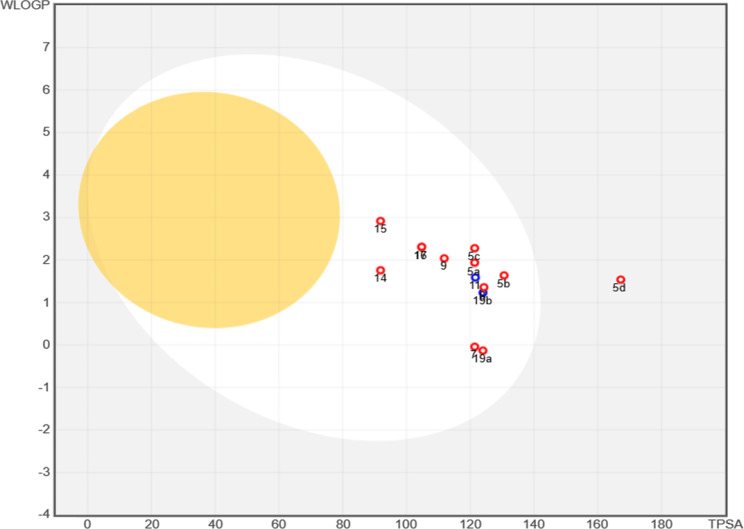
Swiss ADME Boiled-Egg plot showing GI absorption (white) and BBB permeability (yellow); points outside suggest low absorption or brain access

The prepared analogues (**5a–5d**,** 7**,** 8**,** 9**,** 11**,** 14–17**,** 19a**, and **19b**) were estimated by the Swiss ADME online-program to predict tier pharmacokinetic profile (Table 3, and Additional file 1: Fig. S11). Furthermore, the Boiled-Egg plot, as visualized in Fig. 6, provides a complementary assessment, highlighting the relationship between lipophilicity (WLOGP) and topological polar surface area (TPSA) to predict gastrointestinal absorption and blood–brain barrier (BBB) permeability. Compounds **5a**, **5b**, and **5c** displayed excellent pharmacokinetic profiles with high gastrointestinal (GI) absorption, no BBB permeability, and no Pgp substrate properties. Their TPSA ranged from 121.38 to 130.61 Å², with M.wt. between 328.33 and 348.75 g/mol, making them soluble and compliant with Lipinski’s rules. However, **5d** had higher TPSA (167.2 Å²) and low GI absorption, suggesting that its structural bulk reduces its absorption potential. Though, compound **7** showed exceptional properties, with a low M.wt. (238.2 g/mol), very high solubility, high GI absorption, and no BBB permeability or Pgp substrate activity. Its favorable iLOGP (0.73) and low rotatable bonds (1) make it a highly druggable candidate. However, compound **8** exhibited high GI absorption, moderate TPSA (124.28 Å²), and a slightly higher MW (337.36 g/mol), maintaining solubility and drug-likeness. Similarly, compound **9** (M. wt. = 335.38 g/mol) displayed a high GI absorption profile and a lower TPSA (111.84 Å²), indicating good balance for oral bioavailability. Meanwhile, Compound **11** showed high GI absorption but violated Lipinski’s rules due to 4 hydrogen bond donors (HBD), limiting its development as an oral drug. Moreover, compounds **14–17** demonstrated consistent pharmacokinetic properties, with M. wt’s between 266.25 and 317.3 g/mol, TPSA ranging from 91.87 to 104.76 Å², and high GI absorption. All were soluble, BBB impermeant, and non-Pgp substrates, confirming their drug-likeness and favorable oral bioavailability. Furthermore, compound **19a** displayed excellent solubility, a lower M. wt. (270.25 g/mol), and very high GI absorption, making it a strong candidate for oral delivery. In contrast, **19b** (M. wt. = 332.32 g/mol, TPSA = 123.98 Å²) exhibited high GI absorption but was identified as a Pgp substrate, suggesting susceptibility to efflux mechanisms that may reduce its intracellular availability.

**Table 3:** ADME and drug-likeness profiles of the new compounds

Cpd. No.	**MW**	Rotatable bonds	HBA	HBD	TPSA	I LOGP	ESOL Class	GI absorption	BBB permeant	Pgp substrate	Lipinski violations
5a	328.33	2	5	1	121.38	1.66	Soluble	High	No	No	0
5b	344.33	3	6	1	130.61	1.96	Soluble	High	No	No	0
5c	348.75	2	5	1	121.38	2.05	Soluble	High	No	No	0
5d	359.3	3	7	1	167.2	1.53	Soluble	Low	No	No	0
7	238.2	1	5	1	121.38	0.73	Very Soluble	High	No	No	0
8	337.36	4	5	1	124.28	1.54	Soluble	High	No	No	0
9	335.38	4	4	1	111.84	2.05	Soluble	High	No	No	0
11	295.3	5	4	4	121.61	0.52	Soluble	High	No	Yes	0
14	266.25	3	5	2	91.87	1.41	Soluble	High	No	No	0
15	316.31	3	5	2	91.87	1.86	Soluble	High	No	No	0
16	317.3	3	6	2	104.76	1.67	Soluble	High	No	No	0
17	317.3	3	6	2	104.76	1.77	Soluble	High	No	No	0
19a	270.25	3	6	2	123.98	1.01	Very Soluble	High	No	No	0
19b	332.32	4	6	2	123.98	1.9	Soluble	High	No	Yes	0

## Conclusion

In conclusion, a series of novel heterocyclic compounds incorporating 2-pyridone, thiazole, pyrazole, chromene, oxazine, and triazine scaffolds fused with a pyrazine core were successfully synthesized and characterized. Their antibacterial activity was systematically evaluated against both Gram-positive (*S. aureus*, *B. subtilis*) and Gram-negative bacterial strains (*E. coli*, *K. pneumoniae*), and complemented by molecular docking and in silico pharmacokinetic profiling. Among the tested compounds, pyrazine-pyridone derivative **5d** emerged as the most promising candidate, exhibiting strong inhibitory activity against *S. aureus* and *E. coli*, a superior binding affinity with DNA gyrase subunit B (PDB: 4DUH), and favorable docking interactions (S= -7.4519 kcal/mol). Additionally, compound **9** demonstrated notable dual activity against both Gram-positive and Gram-negative strains and showed robust binding and ADME profiles. Moreover, compound **7**, while moderate in bioactivity, displayed exceptional pharmacokinetic properties, including high solubility, low molecular weight, and optimal absorption, suggesting excellent drug-likeness. Collectively, these findings highlight compounds **5d**,** 9**, and **7** as lead candidates for further optimization and preclinical development as potential antibacterial agents.

## Electronic supplementary material

Below is the link to the electronic supplementary material.

Supplementary Material 1

## Data Availability

The datasets used and/or analyzed during the current study are available from the corresponding author on reasonable request.

## Declarations

### Third-party material permissions

All material is original or reproduced with proper permission. No third-party permissions are required.

### Dual publication / prior dissemination

The results/data/figures in this manuscript have not been published elsewhere, nor are they under consideration by another publisher.
