# Supplementary material for: Molecular structures and in Silico molecular docking of new pyrazine-based heterocycles as antibacterial agents
Source: BMC Chem. 2025 Jun 11;19(1):164. doi: 10.1186/s13065-025-01535-w (PMC12153137; doi:10.1186/s13065-025-01535-w)
Supplement: Supplementary file 1 — Supplementary Material 1 [file 13065_2025_1535_MOESM1_ESM.docx]

**Molecular structures and in silico molecular docking of new pyrazine-based heterocycles as antibacterial agents**

Mohamed R. Elmorsy*, Sara H. Yousef, Ehab Abdel-Latif, Safa A. Badawy

*Department of Chemistry, Faculty of Science, Mansoura University, 35516 Mansoura, Egypt,*

^*^Correspondence: [m.r.elmorsy@gmail.com](mailto:m.r.elmorsy@gmail.com)

**Supporting Information**

1. **Experimental general remarks:**

All solvents and chemicals used in this work were analytical grade and used without further purification. Melting points were obtained by the Gallenkamp apparatus. The IR spectra (KBr) were determined on a Thermo Scientific Nicolet iS10 FTIR spectrometer (Faculty of Science, Mansoura University). The ^1^H NMR and ^13^C NMR spectra were recorded by JEOL’s NMR spectrometer (500 MHz) at Faculty of Science, Mansoura University and Bruker NMR spectrometers (400 MHz) in DMSO-d6. The Perkin-Elmer 2400 analyzer was used to obtain the elemental analysis of C, H, and N.

1. **Docking analysis:**

Pyridine derivative **5b** exhibited two hydrogen-acceptor bonds, achieving a noteworthy score of (S = ‐7.3835 kcal/mol) for the bond between the N6-atom of the pyrazine ring and amino acid Lys 103 at an intermolecular distance of 3.23 Å, as well as between the N24-atom nitrile group and Gly 77 with a root-mean-square deviation (RMSD =1.1571)). In contrast, pyridine analogues **5a** and **5c** showed similar interactions arising from two intermolecular attractions, which included two hydrogen-donor bonds involving the N20, N21 atoms of the amino group and Asp 73 (2.90 Å, 2.91 Å) and two π-hydrogen bonds between the pyrid-2-one ring and benzene ring with Asn 46, with binding scores (S = ‐7.3018 and S = ‐7.2467 kcal/mol), and RMSD (0.6638 and 1.5719), respectively. Additionally, pyridine derivative **5d** displayed a higher binding (S = ‐7.4519 kcal/mol), RMSD = 1.2498 attributed to its two interactions: one hydrogen-donor bond involving the N20-atom of the amino group and Gly 101 (2.95 Å), and another π-hydrogen interaction from the benzene ring with Asn 46 (3.66 Å). Meanwhile, pyridine analogue **7** achieved the lowest binding score (S = ‐6.2950 kcal/mol), with one hydrogen-donor bond between the C10-atom of the pyridine ring and Asp 73, two hydrogen-acceptor interactions, one between the N6-atom of the pyrazine ring and Asp 73, and one from the N16 nitrile group with Gly 77 at intermolecular distances of 3.11 and 2.99 Å, respectively. A fourth attraction was identified as a π-H interaction between the pyrazine ring and Lys 103 (3.94 Å). Furthermore, thiazole derivative **8** showed a binding free energy (S = ‐6.9222 kcal/mol) resulting from a hydrogen-donor bond between the C14-atom of the thiazole ring and Gly 117 (3.18 Å) and a hydrogen-acceptor bond between the N18-atom of the nitrile group and Ser 121 (3.67 Å), and an RMSD (1.1637). Thiazole derivative **9** demonstrated a π-H bond between the pyrazine ring and Lys 103 (3.88 Å), achieving a significant docking score (S = ‐7.1973 kcal/mol). Pyrazole derivative **11** exhibited a moderate binding (S = ‐6.9026 kcal/mol), RMSD (1.3146), attributed to a hydrogen-donor bond between the N12-atom of the pyrazole ring and Gly 101. Additionally, analogue **14** attained a weak binding (S = ‐6.7530 kcal/mol), RMSD (1.8810) due to a hydrogen-acceptor bond between the N6-atom of the pyrazine ring and Lys at a distance =1.8810 Å. Meanwhile, analogue **15** showed a hydrogen-acceptor bond with a notable score (S = ‐7.3325 kcal/mol) between the O11-atom of the carbonyl group and Val 120, and RMSD (1.0317). Moreover, oxazine analogues **16** and **17** displayed proper binding scores (S = ‐7.3501 and ‐7.0912 kcal/mol), RMSD (1.3130 and 1.3343), respectively. Analogue **16** exhibited a hydrogen-acceptor bond between the N3-atom of the pyrazine ring and Asn 46, with bond lengths = 3.58 Å. However, analogue **17** demonstrated an interaction between the benzene ring and the Ile 78 amino acid *via* a π-H bond, with a docking score (S= ‐7.0912 kcal/mol), RMSD (1.3343), and a bond length = 1.3343 Å. Lastly, triazine derivatives **19a** and **19b** were analyzed, with derivative **19a** showing four π-H bonds: the first between the pyrazole ring and Asn 46 (3.99 Å), two interactions from the triazine ring with Asn 46 and Ile 78 at distances of 3.71 and 3.73 Å, respectively, and a fourth attraction between the pyrazole ring and Pro 79 with a binding score (S = ‐6.7903 kcal/mol). Meanwhile, derivative **19b** exhibited a hydrogen-donor bond at an intermolecular distance of 2.99 Å between the N11-atom of the triazine ring and Gly 101, along with two π-H bonds involving the pyrazole ring and the triazine ring with Lys 103 (4.10 and 3.75 Å, respectively) and an optimal binding energy (S = ‐7.4427 kcal/mol).


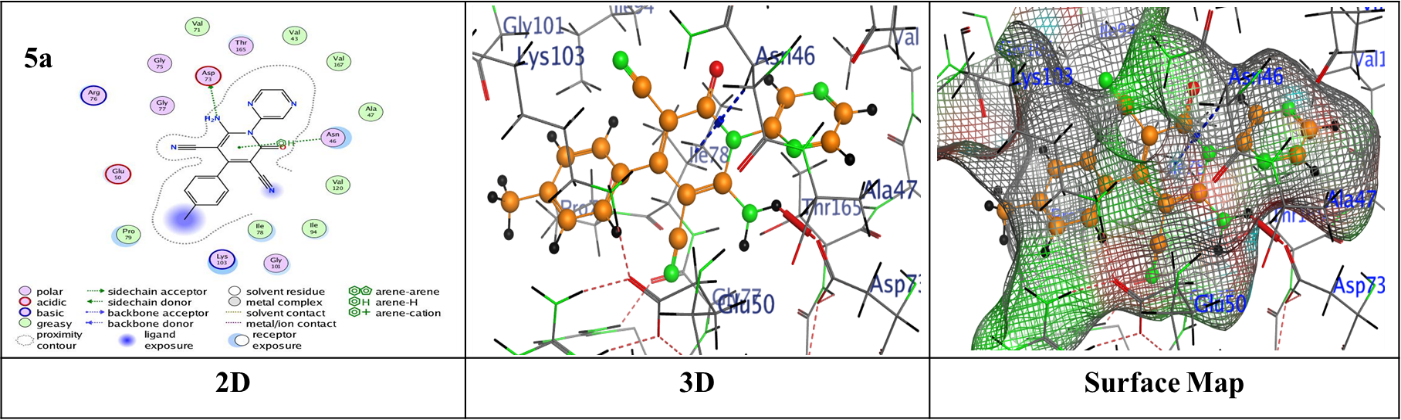


**Fig. S1** The interactions of compound **5a** with active sites of (PDB ID: 4DUH)


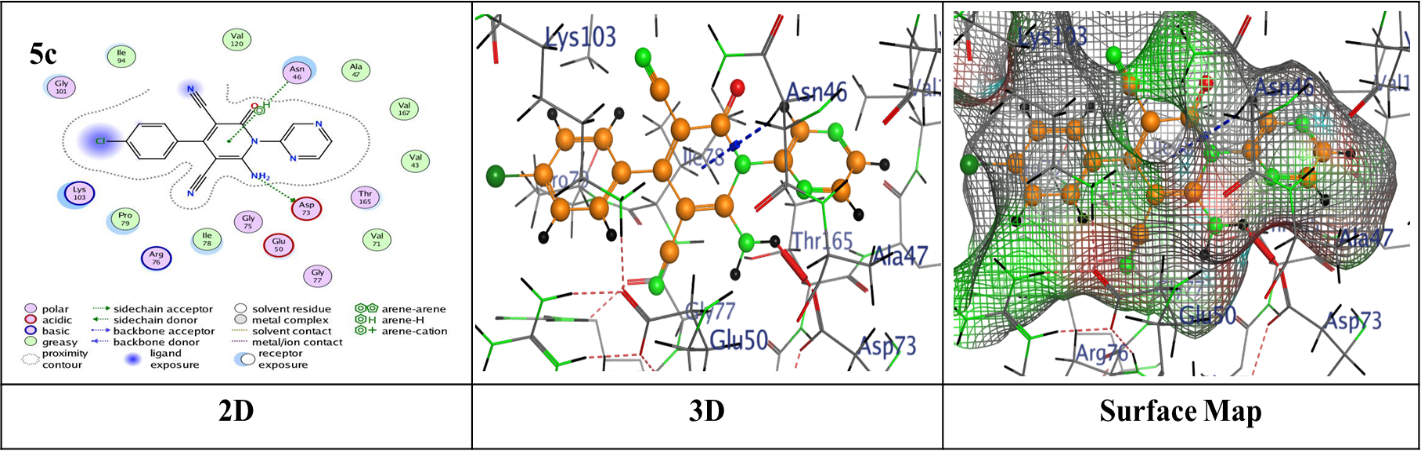
 **Fig. S2** The interactions of compound **5c** with active sites of (PDB ID: 4DUH)


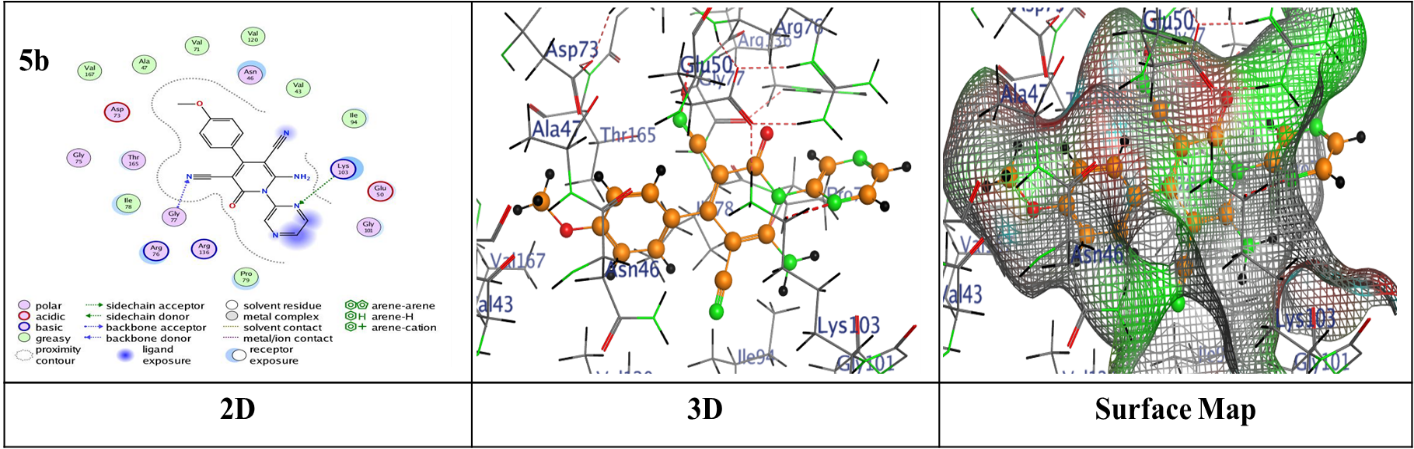
 **Fig. S3** The interactions of compound **5b** with active sites of (PDB ID: 4DUH)


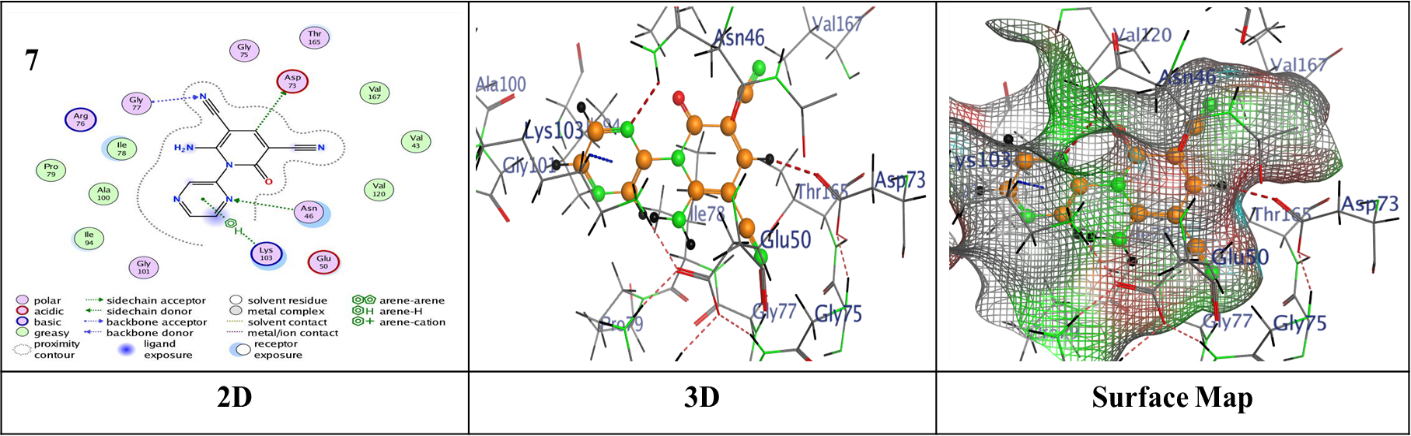


**Fig. S4** The interactions of compound **7** with active sites of (PDB ID: 4DUH)


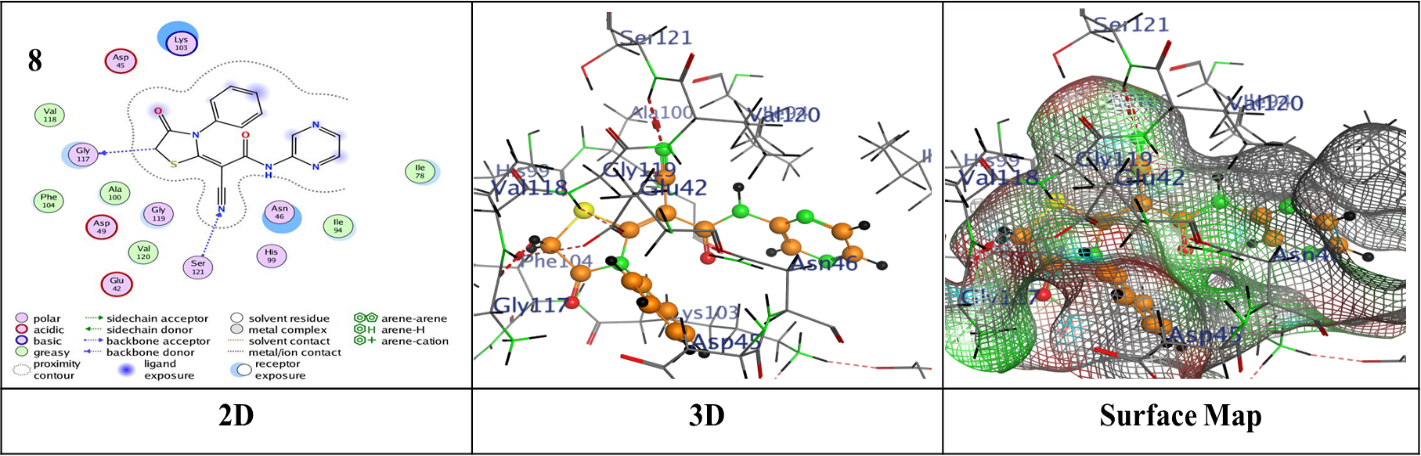


**Fig. S5** The interactions of compound **8** with active sites of (PDB ID: 4DUH)


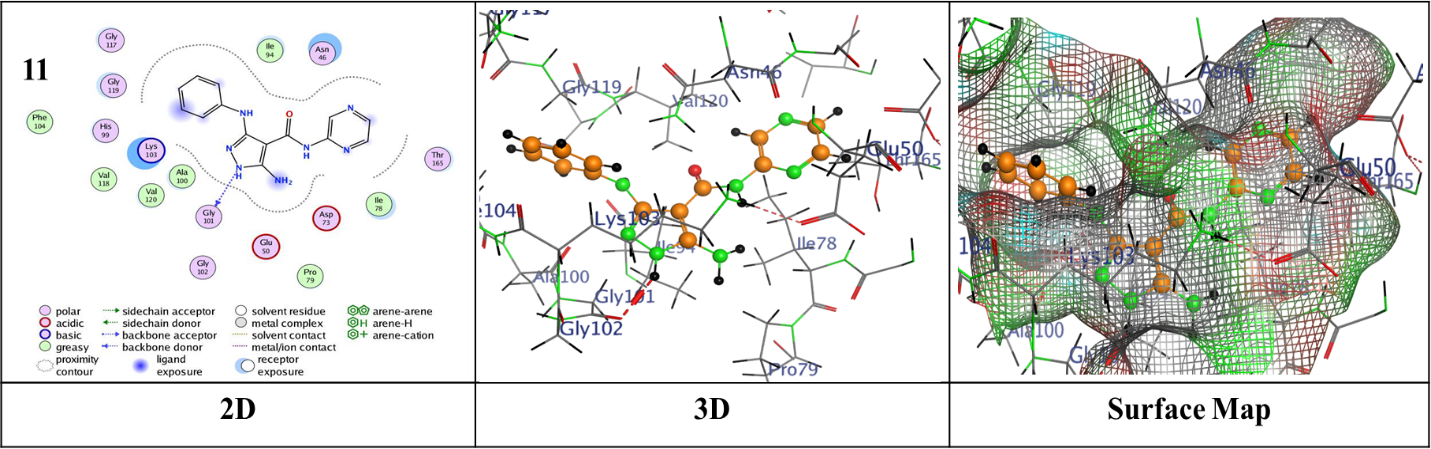


**Fig. S6** The interactions of compound **11** with active sites of (PDB ID: 4DUH)


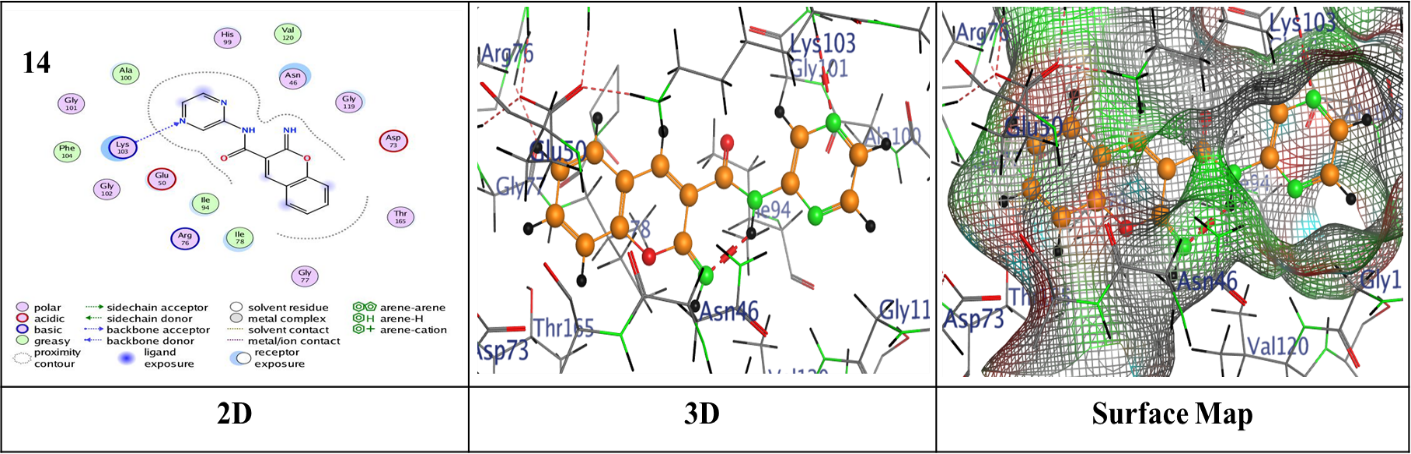


**Fig. S7** The interactions of compound **14** with active sites of (PDB ID: 4DUH)


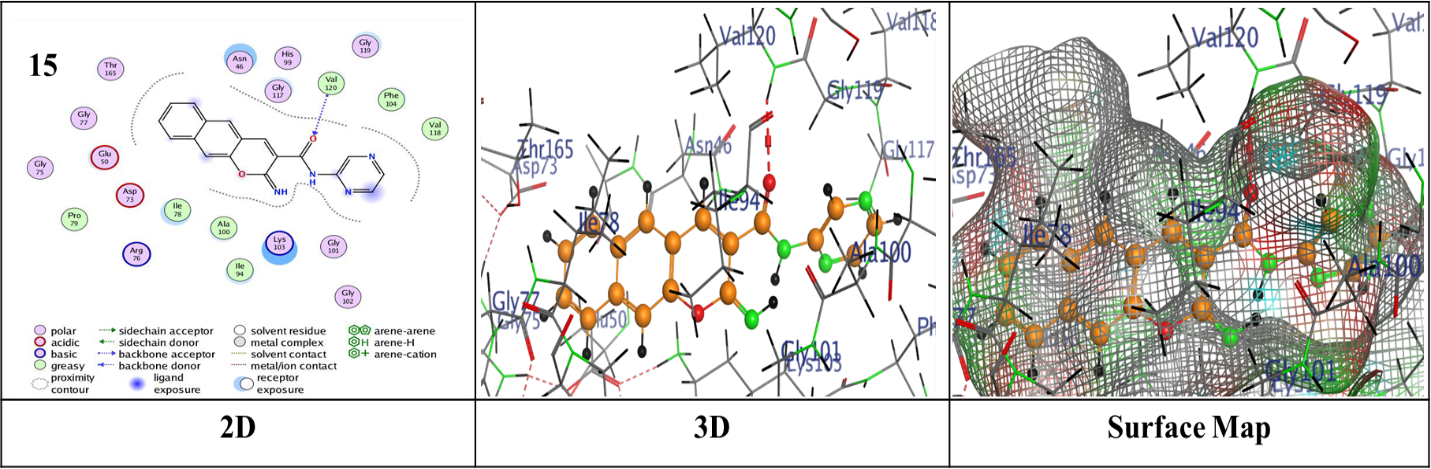


**Fig. S8** The interactions of compound **15** with active sites of (PDB ID: 4DUH)


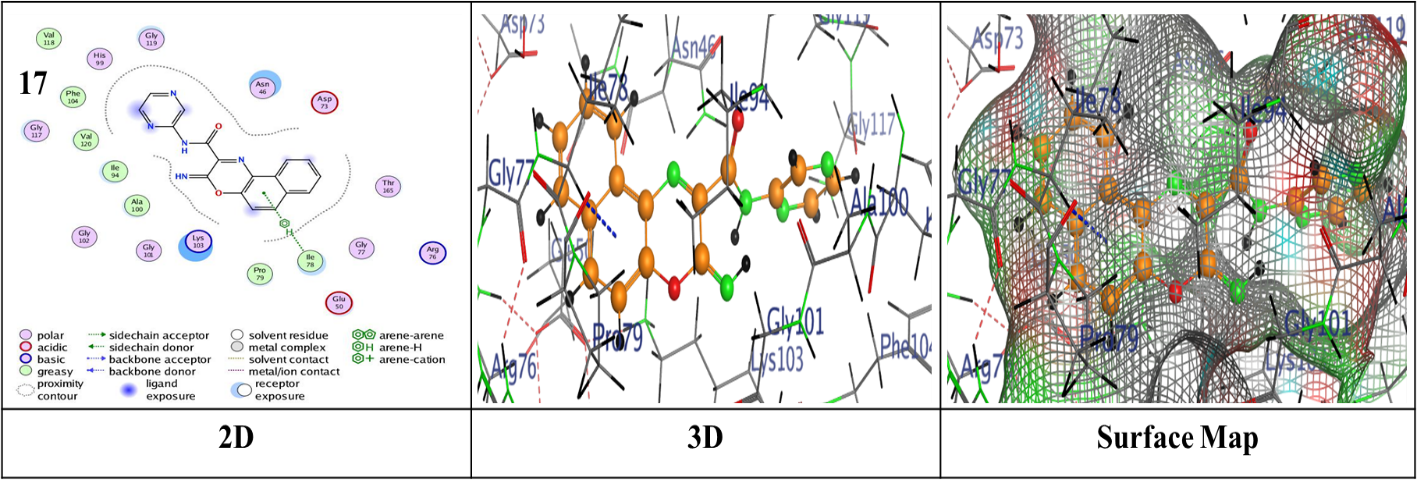


**Fig. S9** The interactions of compound **17** with active sites of (PDB ID: 4DUH)


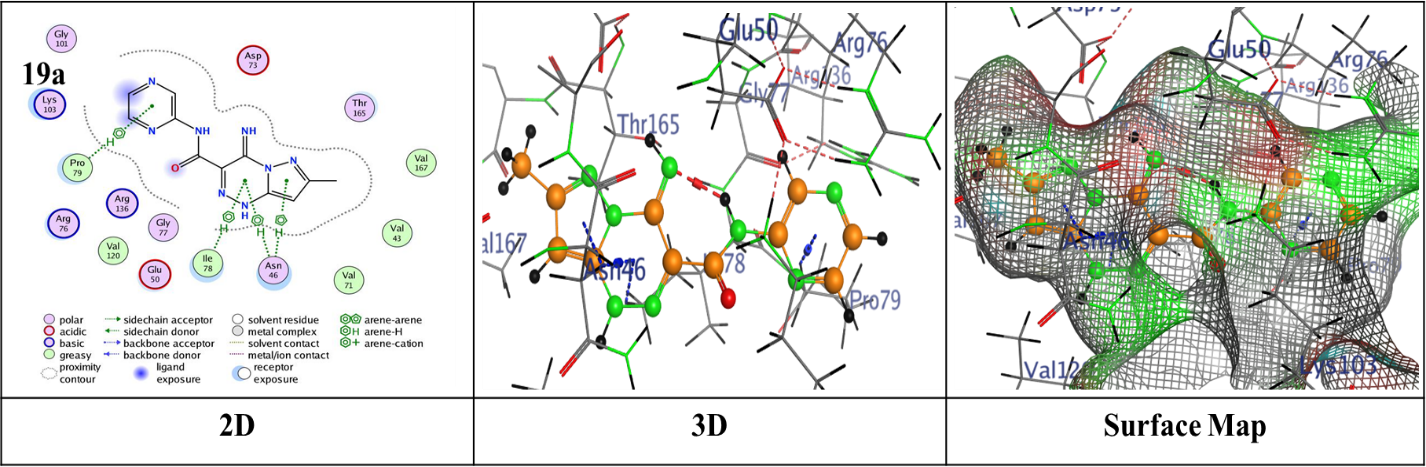


**Fig. S10** The interactions of compound **19a** with active sites of (PDB ID: 4DUH)

1. **Swiss ADME analysis**


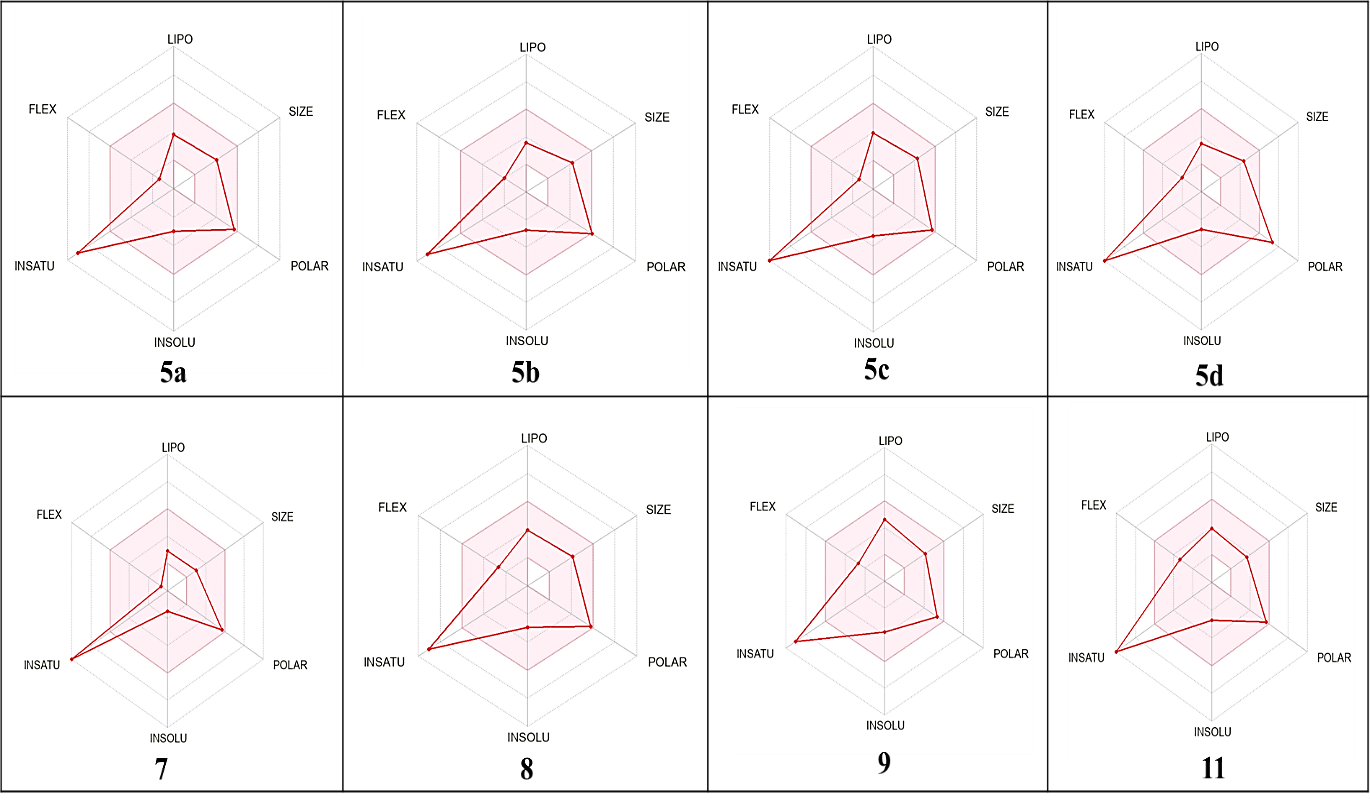


**(Fig. S11a)** Radar Chart of compounds **5a-11**


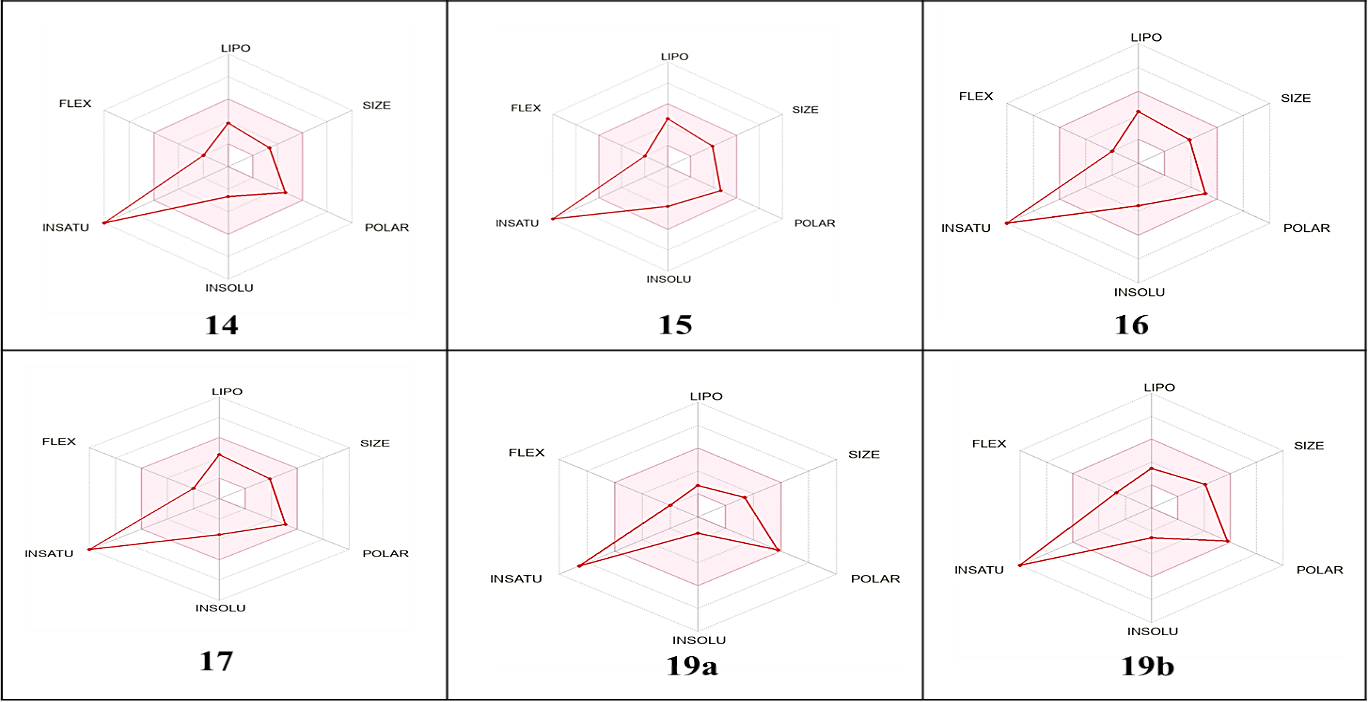


**(Fig. S11b)** Radar Chart of compounds **14-19b**.

1. **Spectral analysis:**


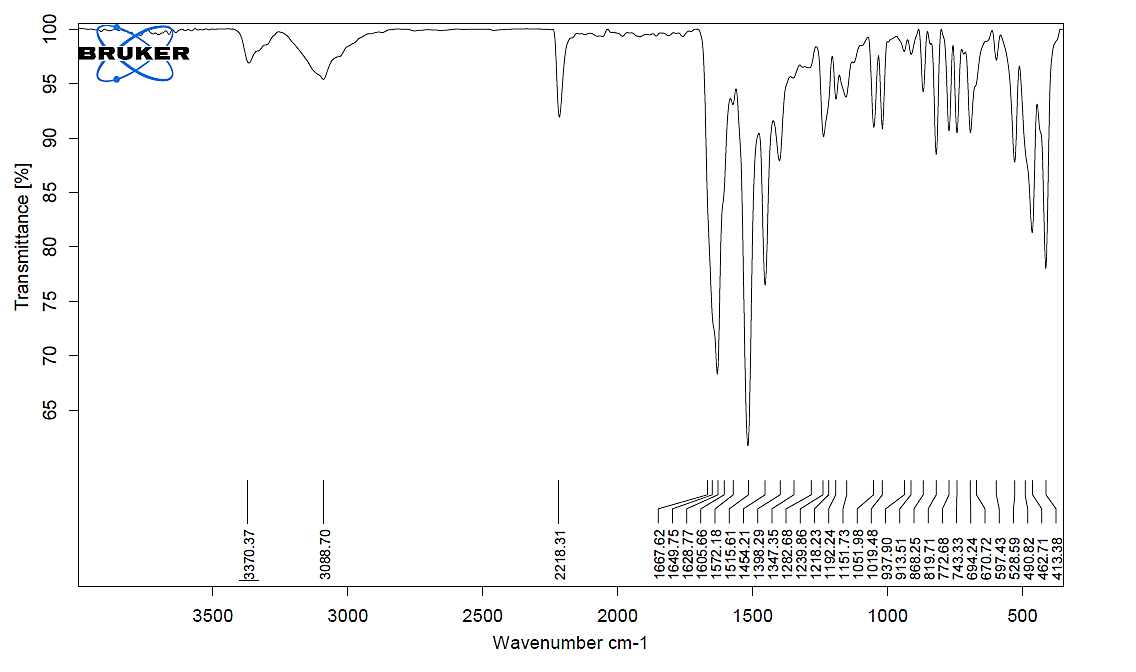

**Figure (S12): IR spectrum of compound 5a**


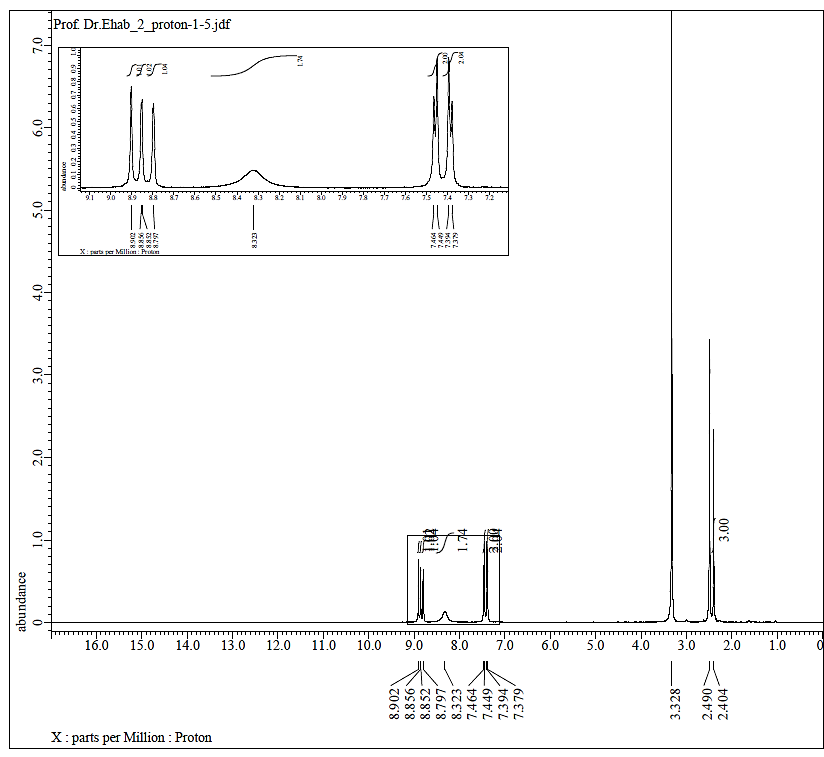

**Figure (S13): ^1^H NMR spectrum of compound 5a**

**
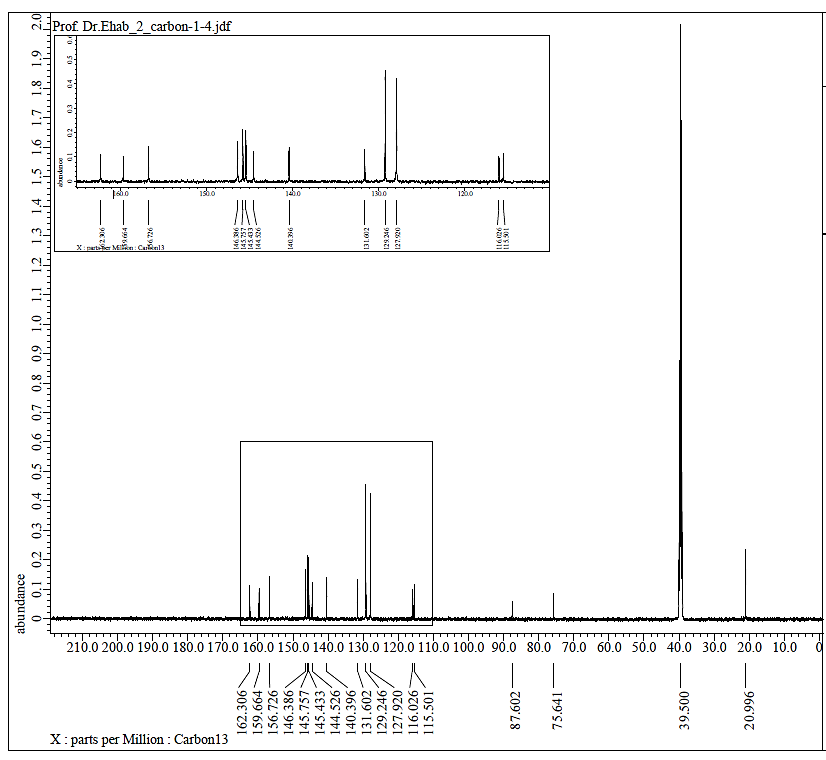
**

**Figure (S14): ^13^C NMR spectrum of compound 5a.**


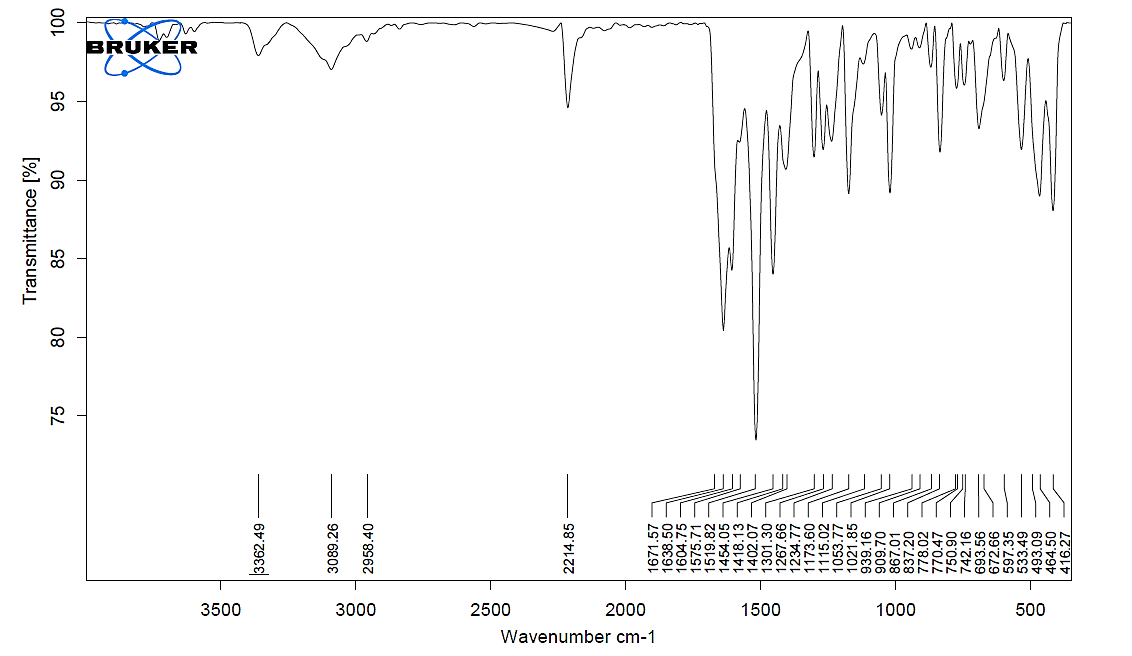

**Figure (S15): IR spectrum of compound 5b.**

**
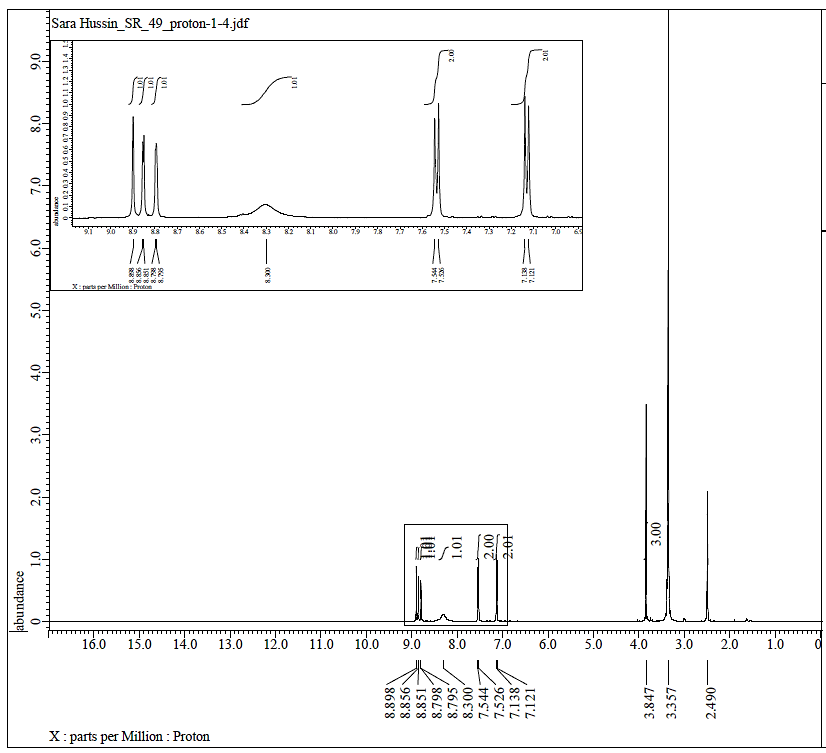
**

**Figure (S16): ^1^H NMR spectrum of compound 5b.**

**
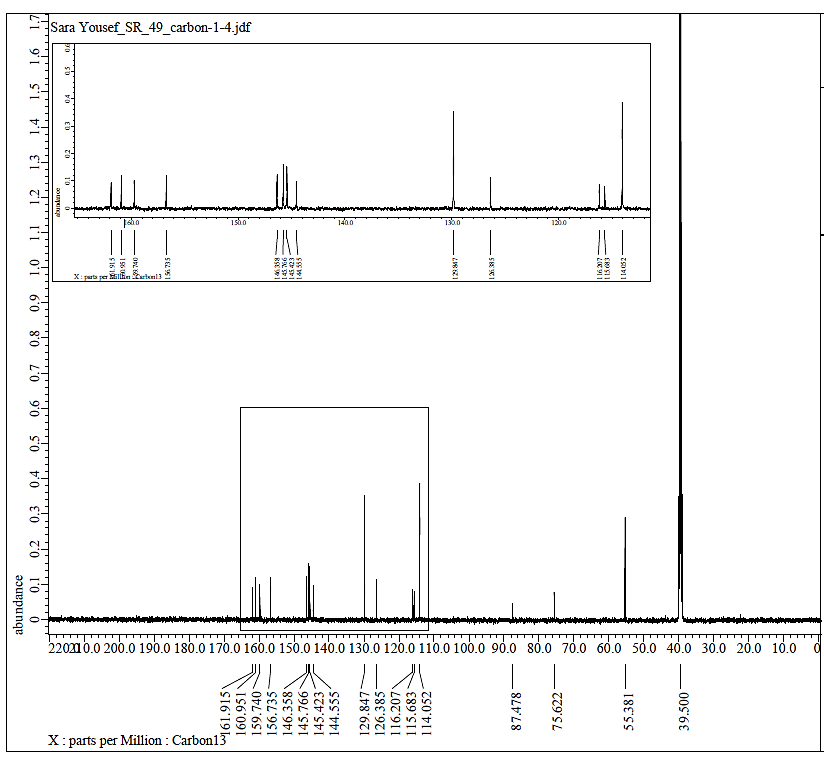
**

**Figure (S17): ^13^C NMR spectrum of compound 5b.**

**
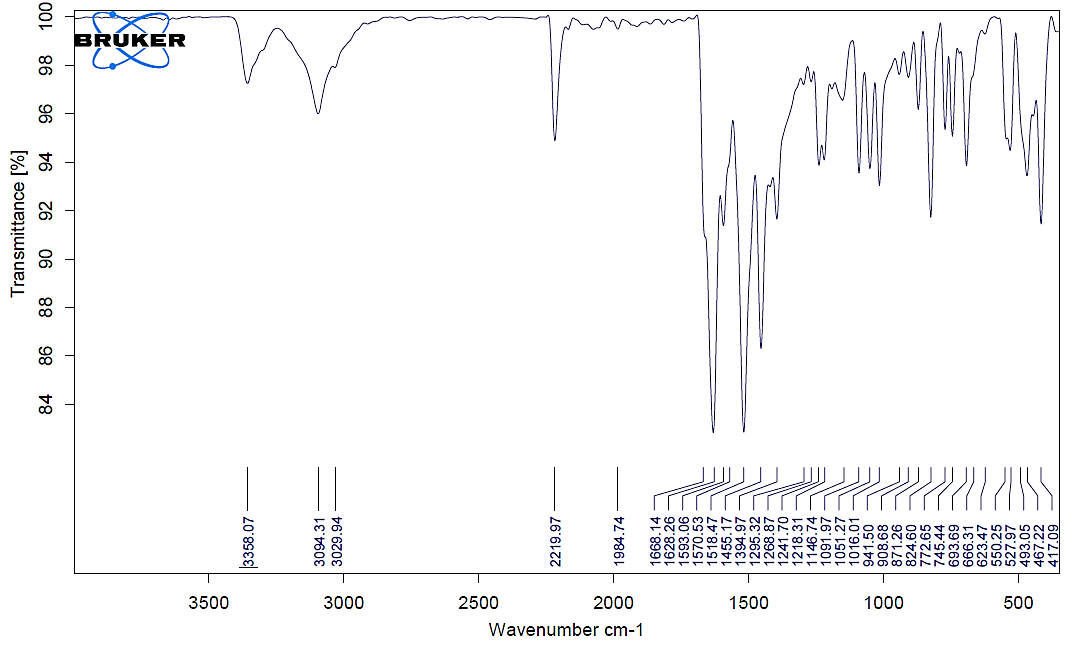
**

**Figure (S18): IR spectrum of compound 5c.**

**
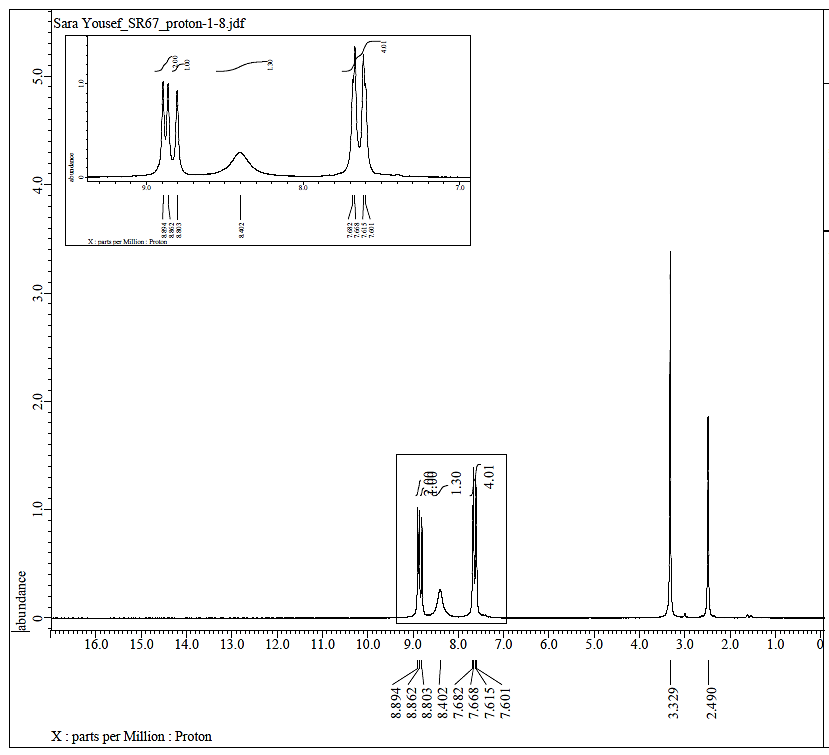
**

**Figure (S19): ^1^H NMR spectrum of compound 5c.**

**
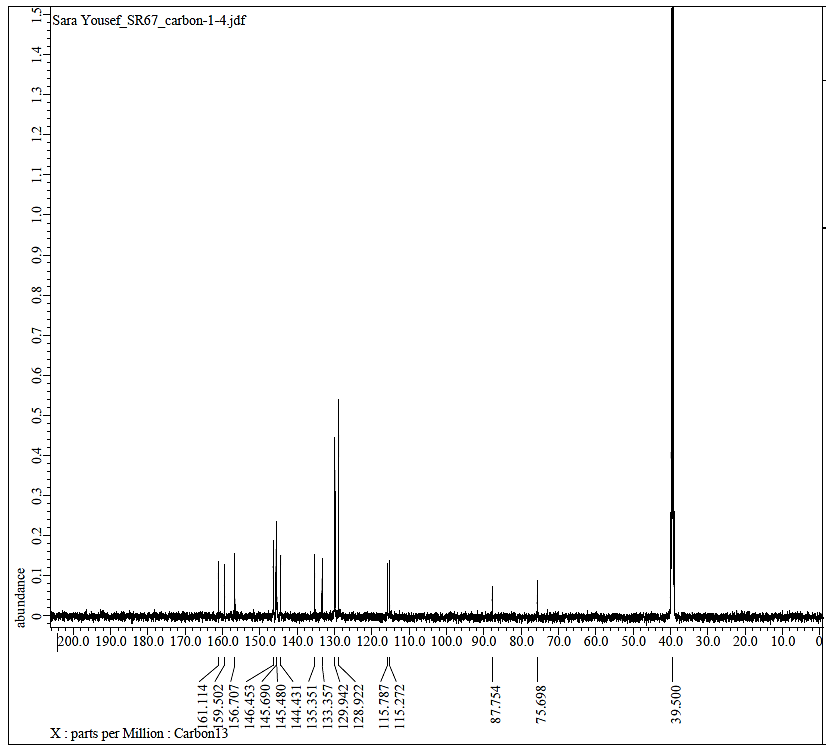
**

**Figure (S20): ^13^C NMR spectrum of compound 5c.**

**
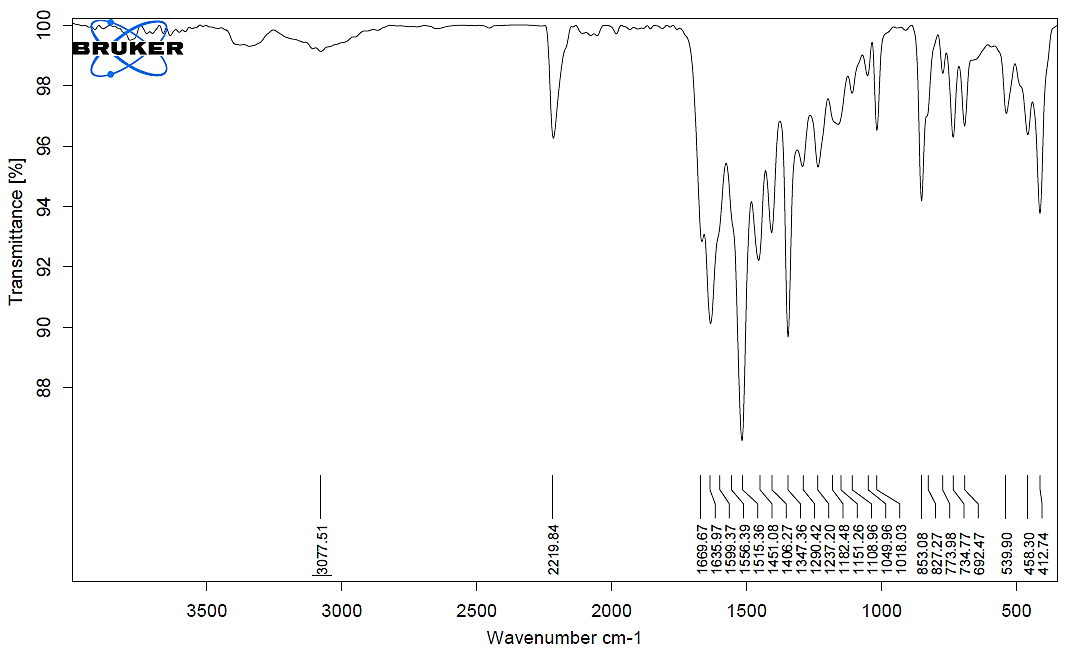
**

**Figure (S21): IR spectrum of compound 5d.**

**
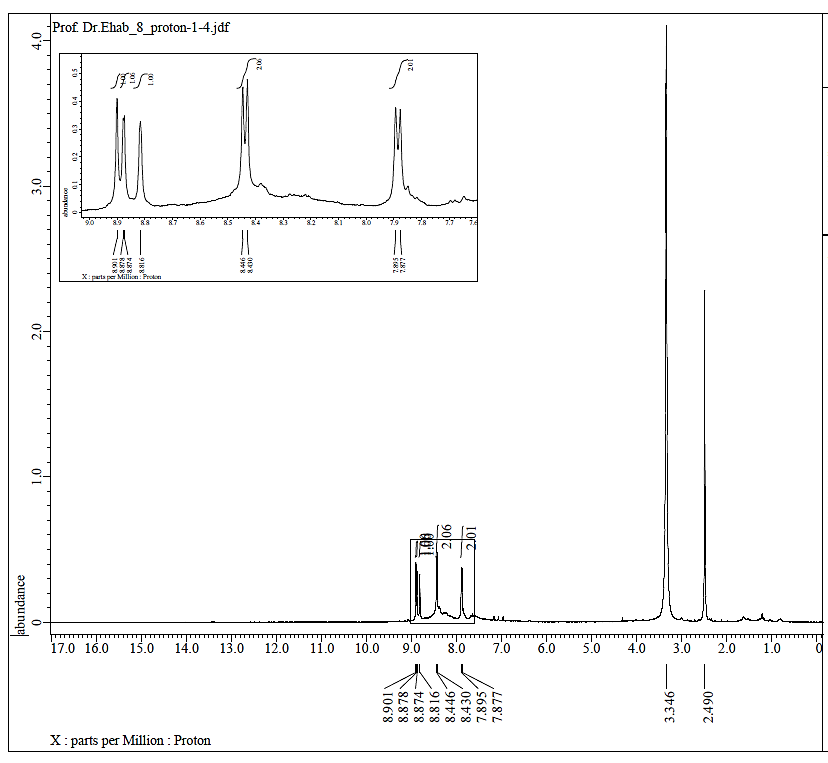
**

**Figure (S22): ^1^H NMR spectrum of compound 5d.**

**
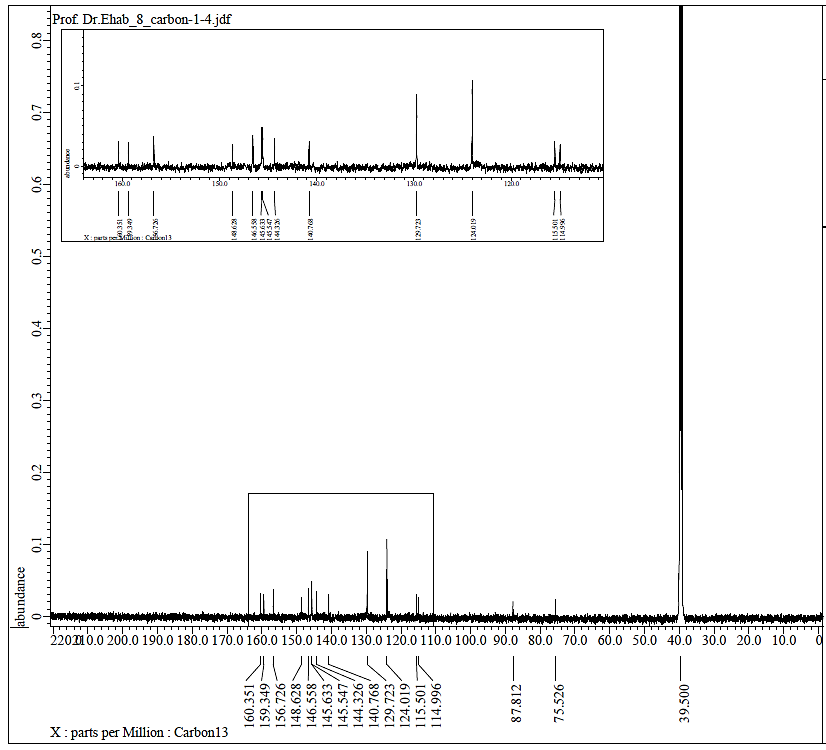
**

**Figure (S23): ^13^C NMR spectrum of compound 5d.**

**
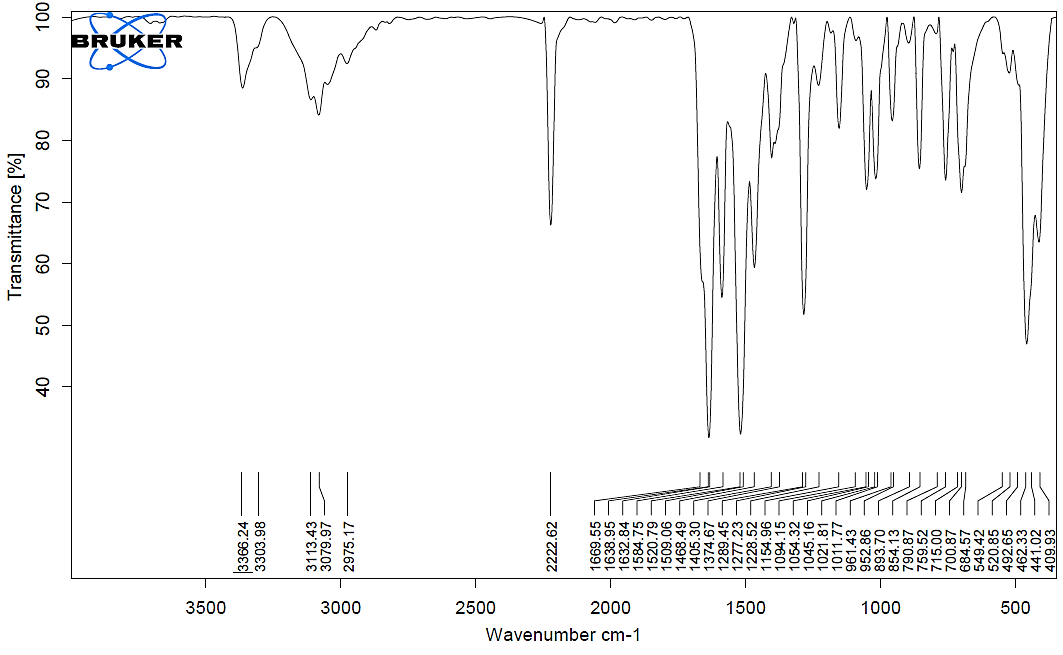
**

**Figure (S24): IR spectrum of compound 7.**

**
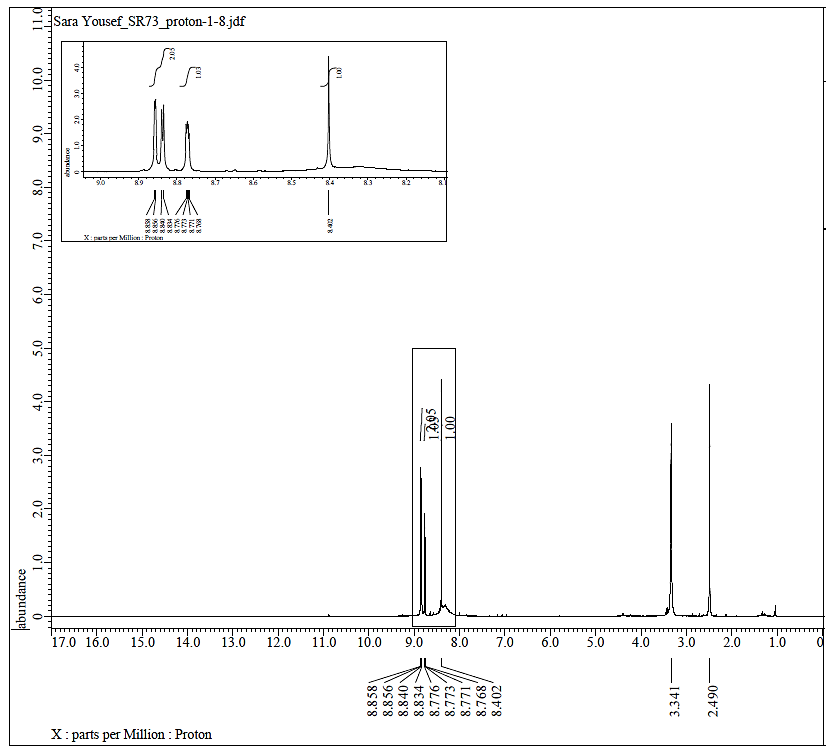
**

**Figure (S25): ^1^H NMR spectrum of compound 7.**

**
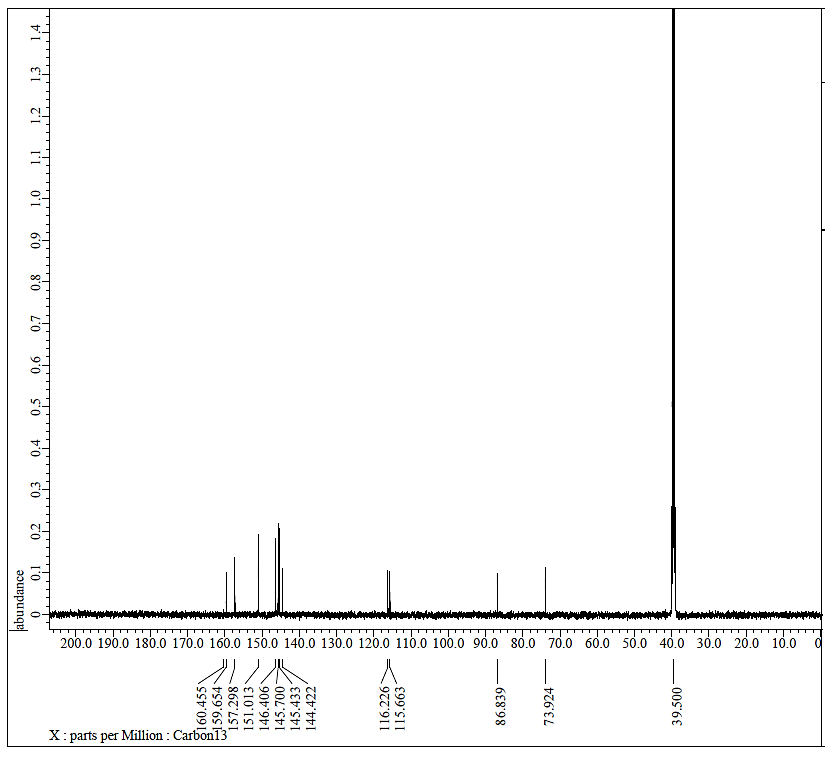
**

**Figure (S26): ^13^C NMR spectrum of compound 7.**

**
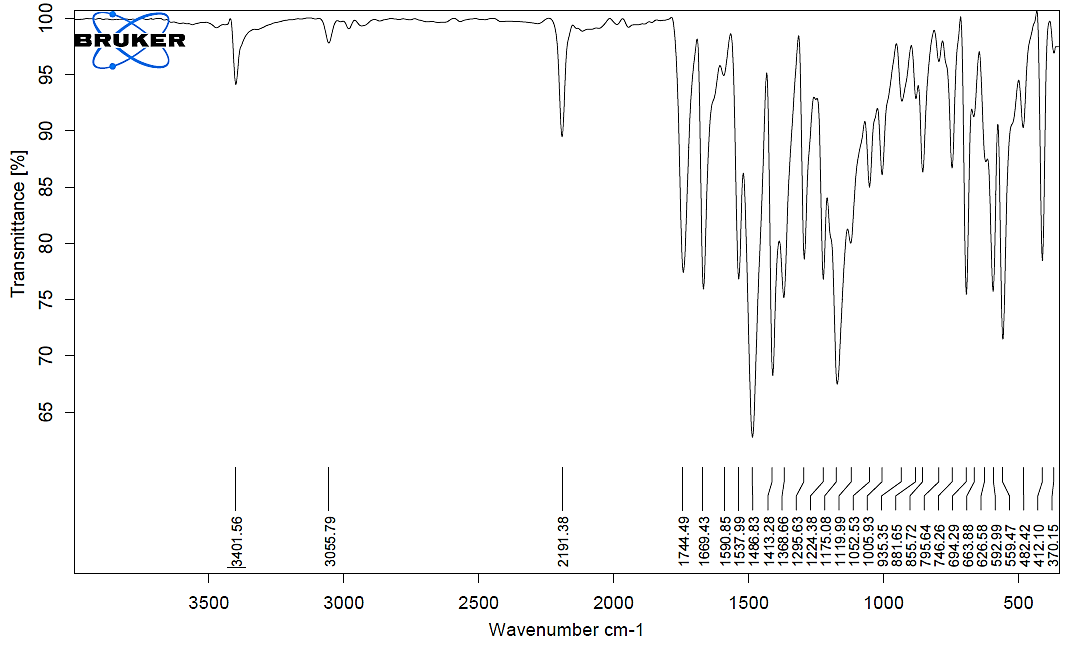
**

**Figure (S27): IR spectrum of compound 8.**

**
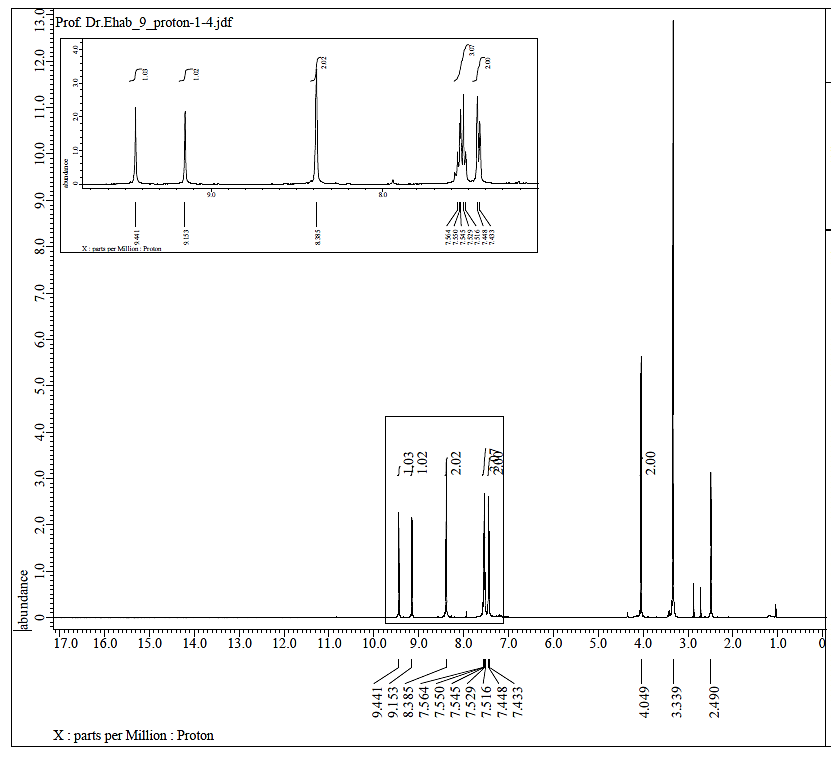
**

**Figure (S28): ^1^H NMR spectrum of compound 8.**

**
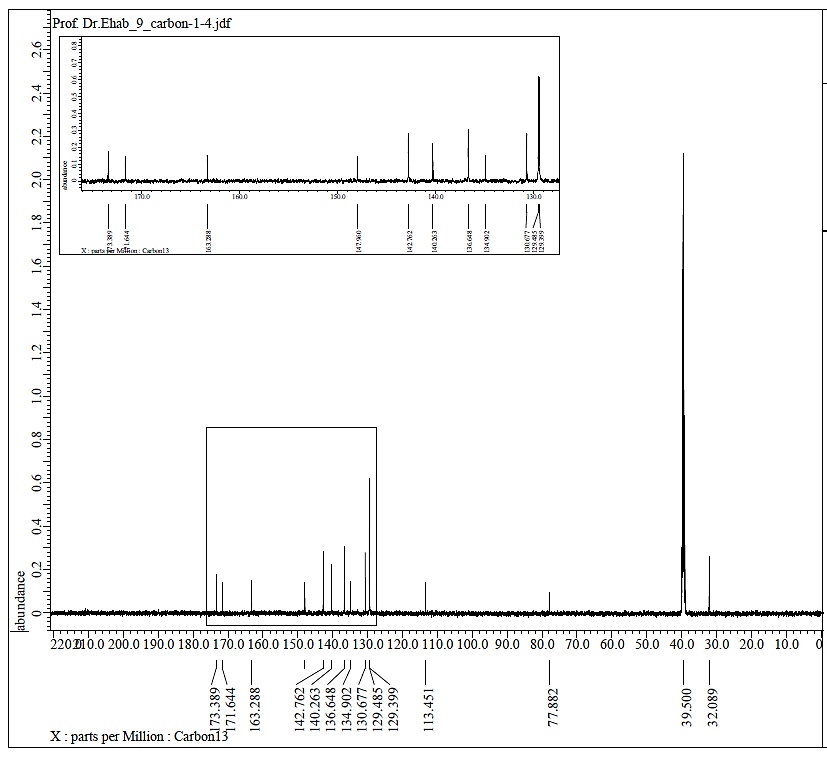
**

**Figure (S29): ^13^C NMR spectrum of compound 8.**

**
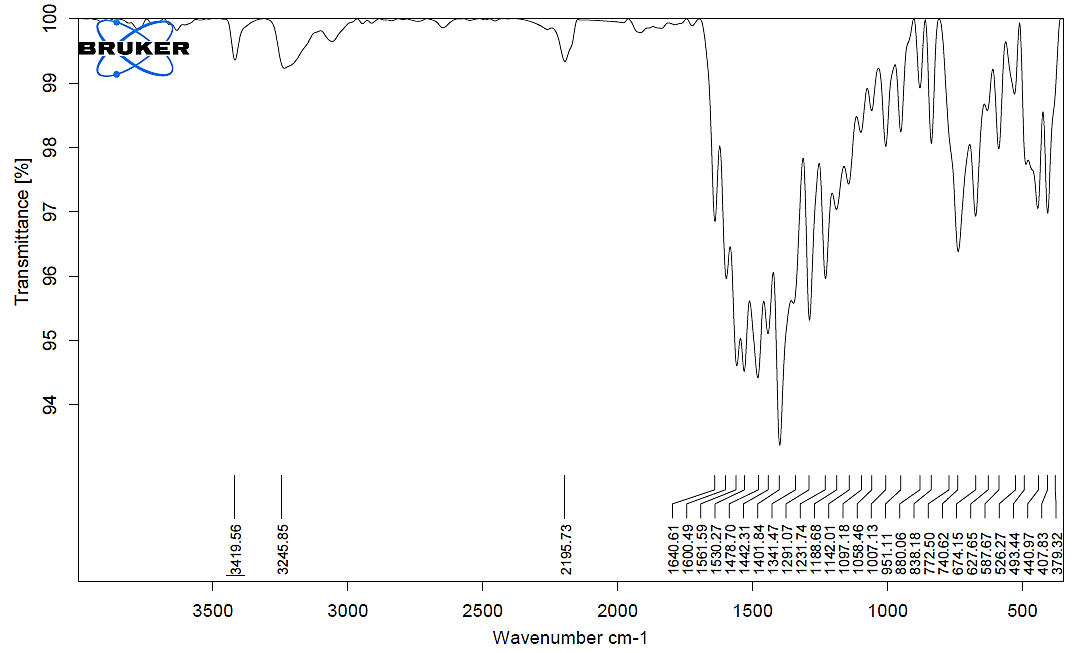
**

**Figure (S30): IR spectrum of compound 9.**

**
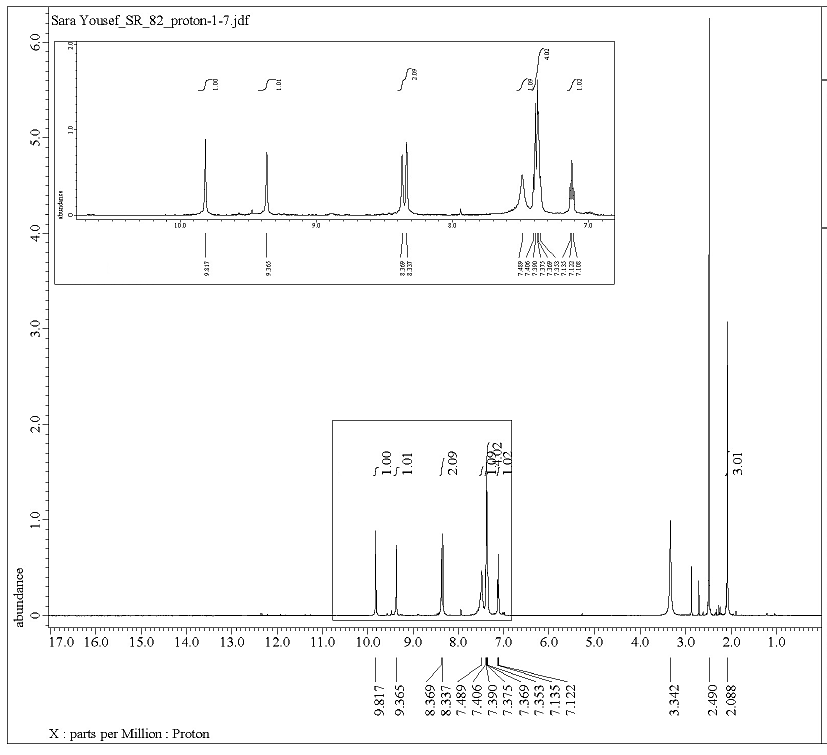
**

**Figure (S31): ^1^H NMR spectrum of compound 9.**

**
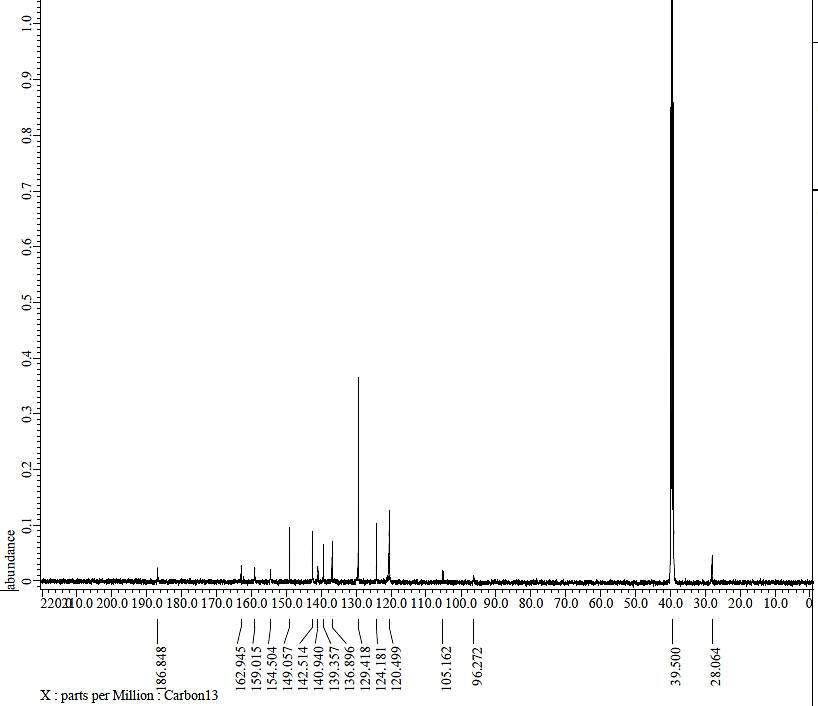
**

**Figure (S32): ^13^C NMR spectrum of compound 9.**

**
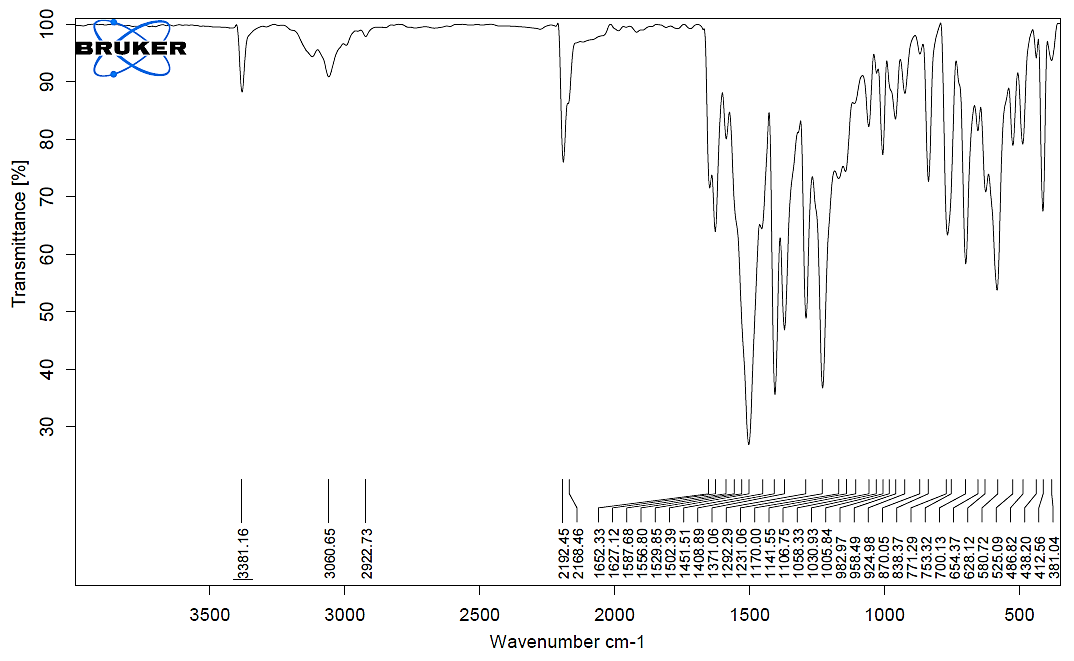
**

**Figure (S33): IR spectrum of compound 10.**

**
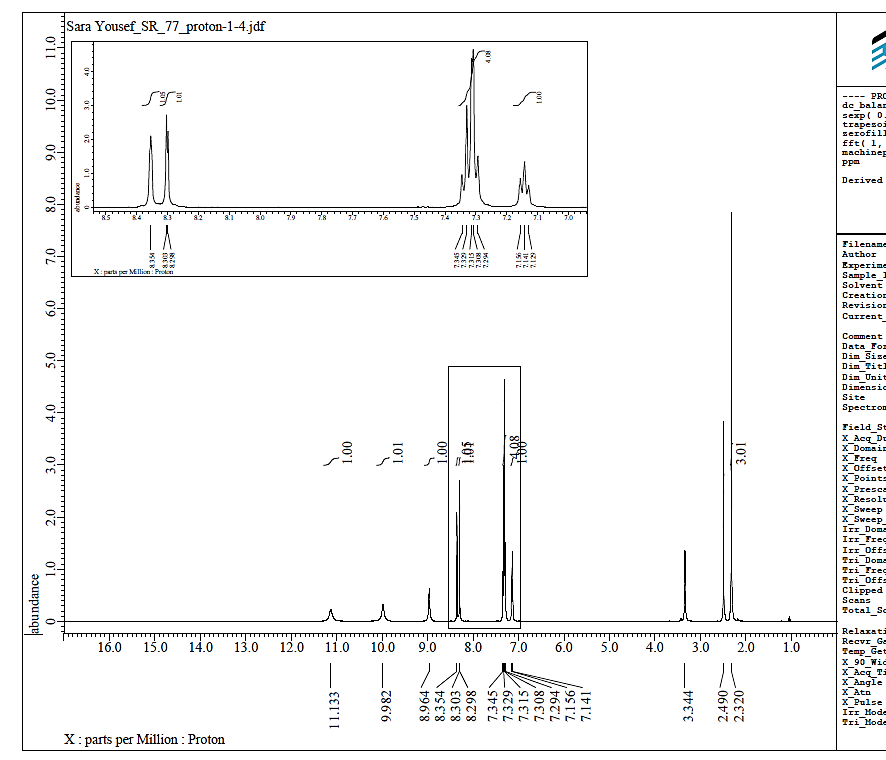
**

**Figure (S34): ^1^H NMR spectrum of compound 10.**

**
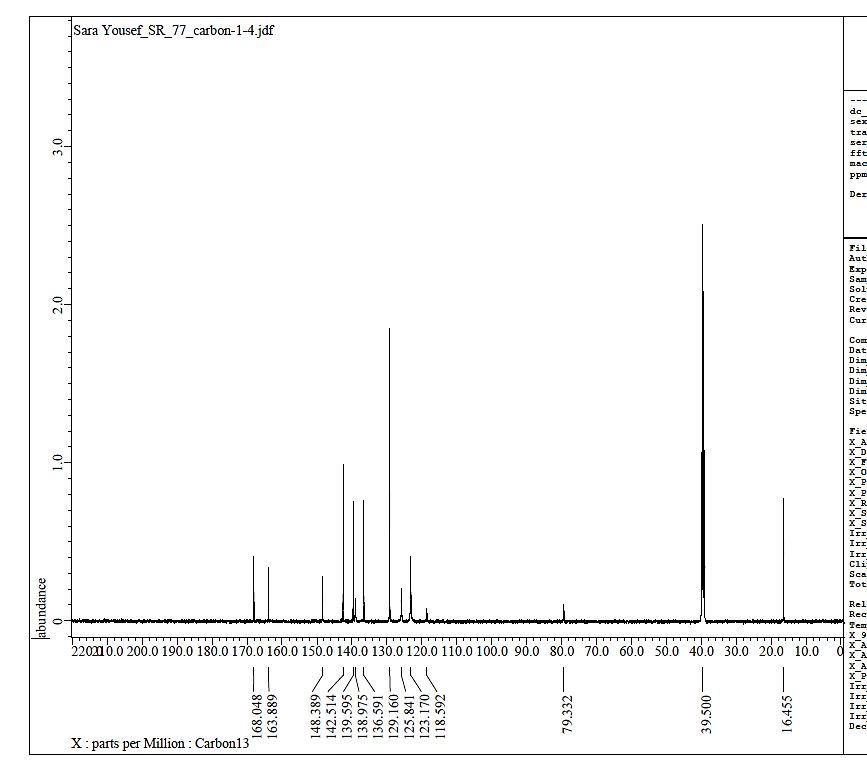
**

**Figure (S35): ^13^C NMR spectrum of compound 10.**

**
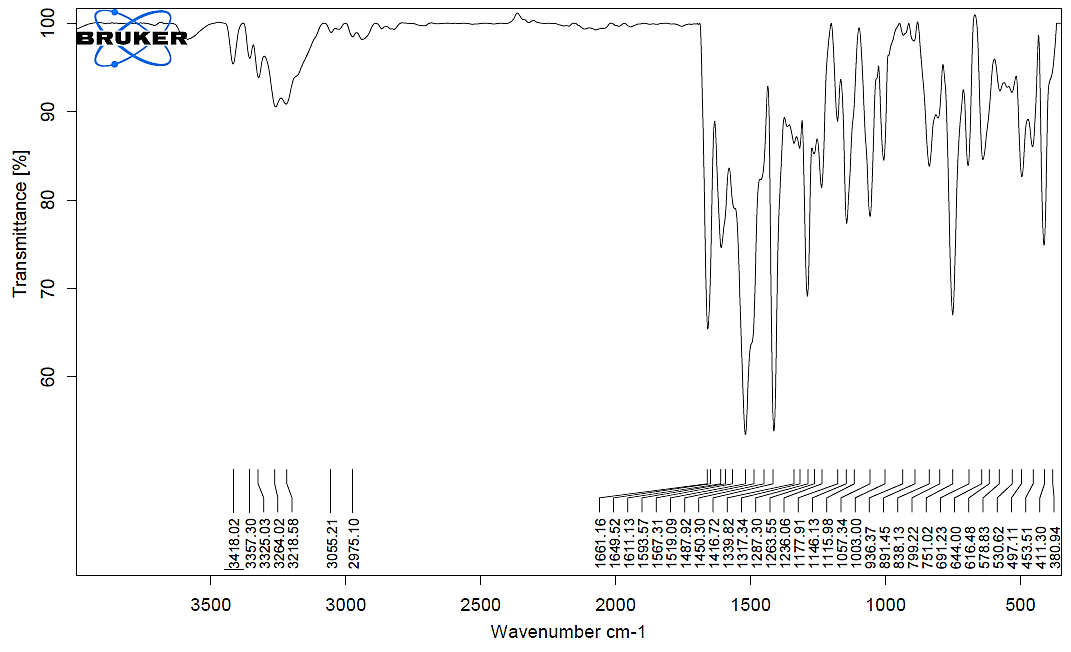
**

**Figure (S36): IR spectrum of compound 11.**

**
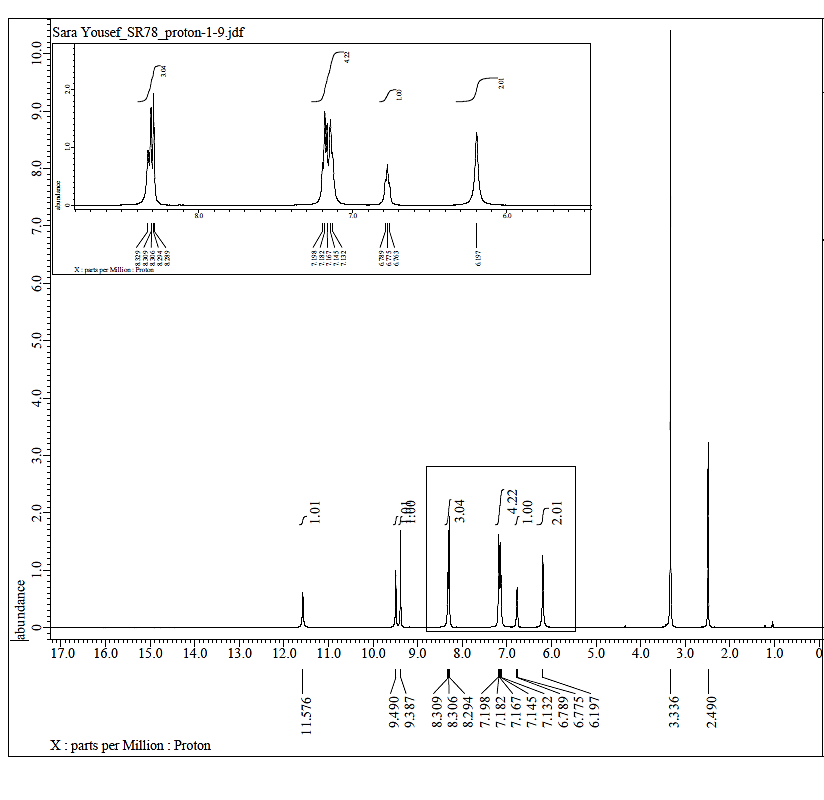
**

**Figure (S37): ^1^H NMR spectrum of compound 11.**

**
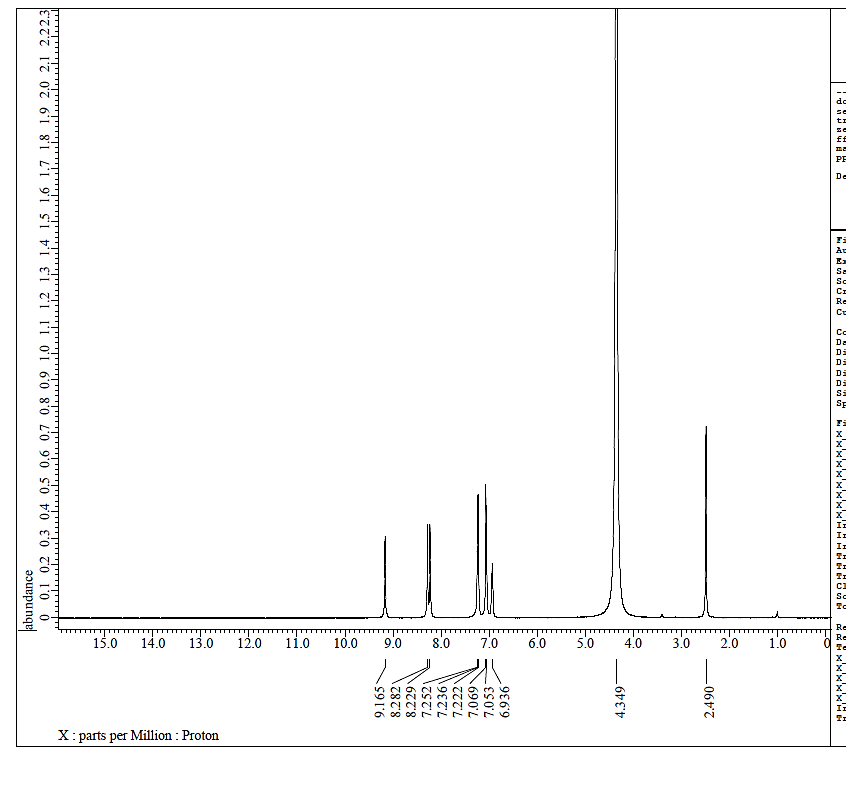
**

**Figure (S38): (D_2_O) ^1^H NMR spectrum of compound 11.**

**
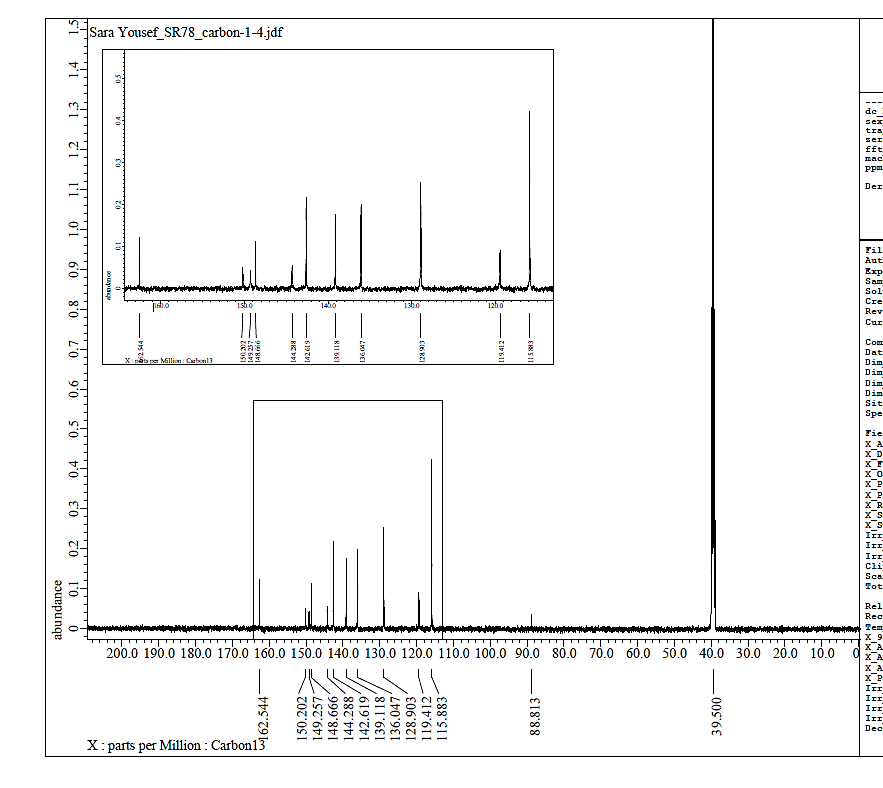
**

**Figure (S39): ^13^C NMR spectrum of compound 11.**

**
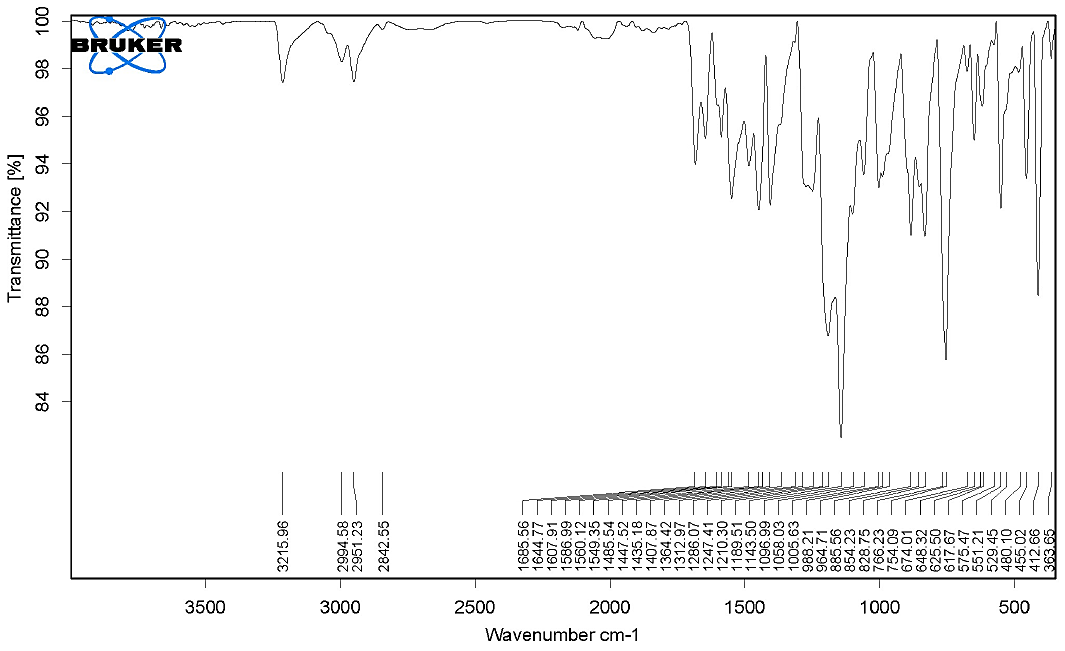
**

**Figure (S40): IR spectrum of compound 14.**

**
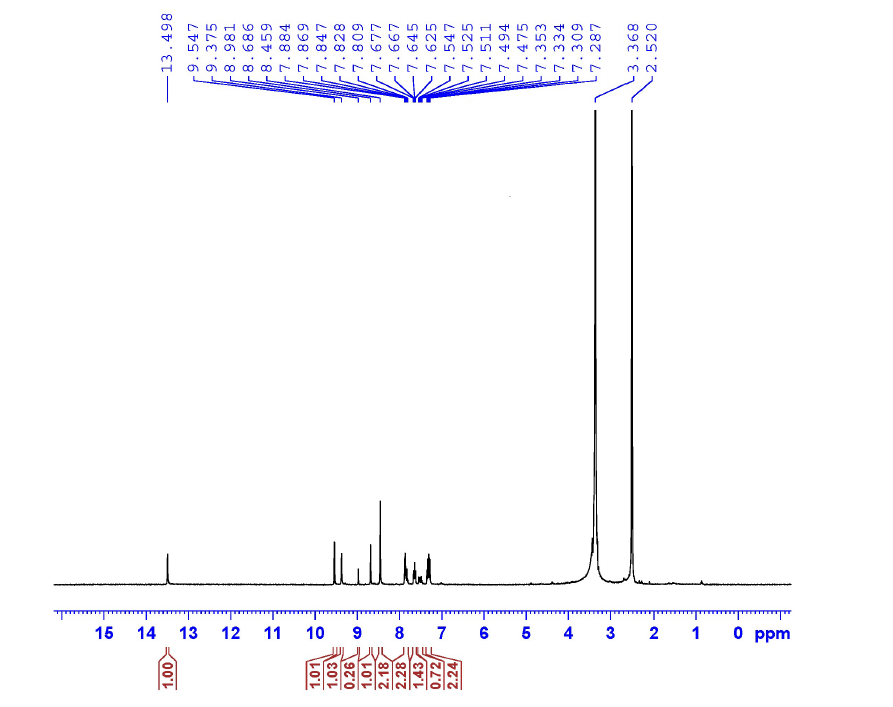
**

**Figure (SS41): ^1^H NMR spectrum of compound 14.**

**
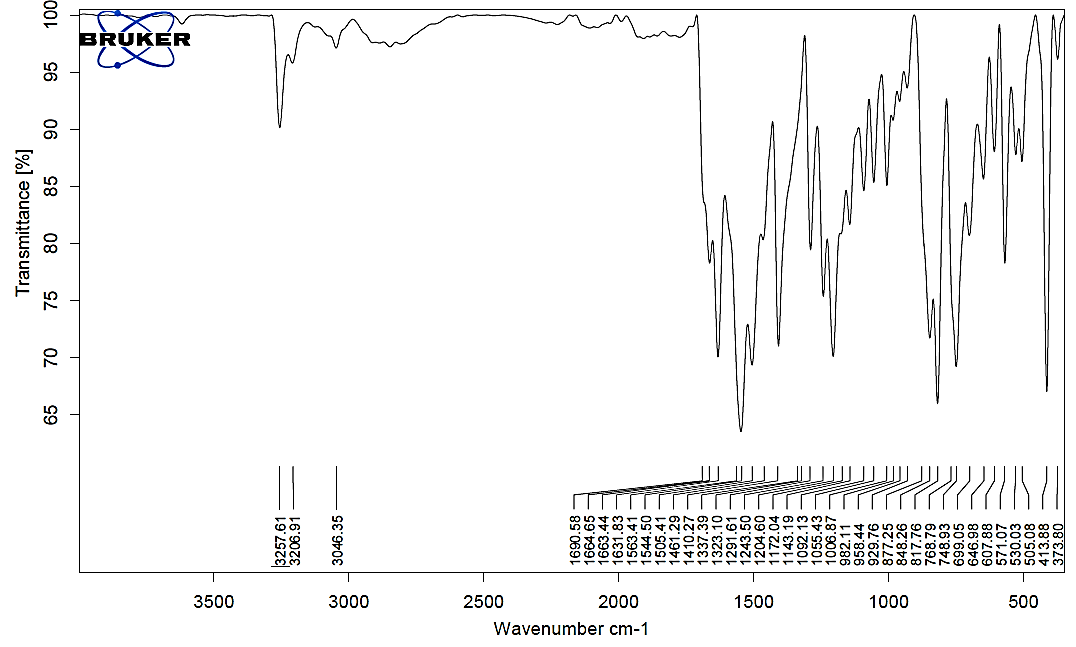
**

**Figure (S42): IR spectrum of compound 15.**

**
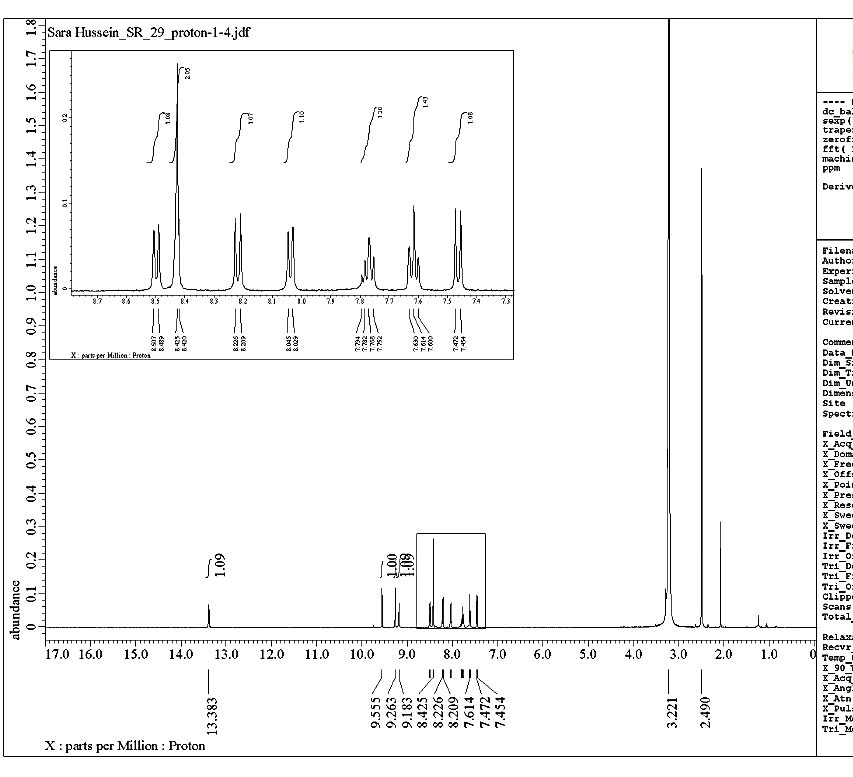
**

**Figure (S43): ^1^H NMR spectrum of compound 15.**

**
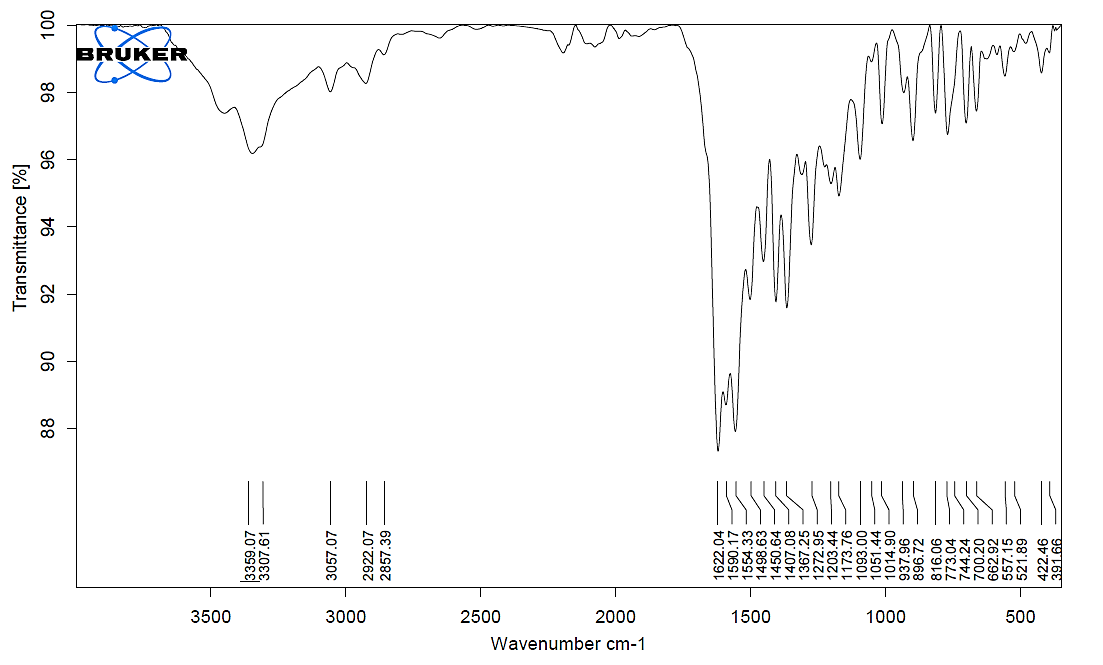
**

**Figure (S44): IR spectrum of compound 16.**

**
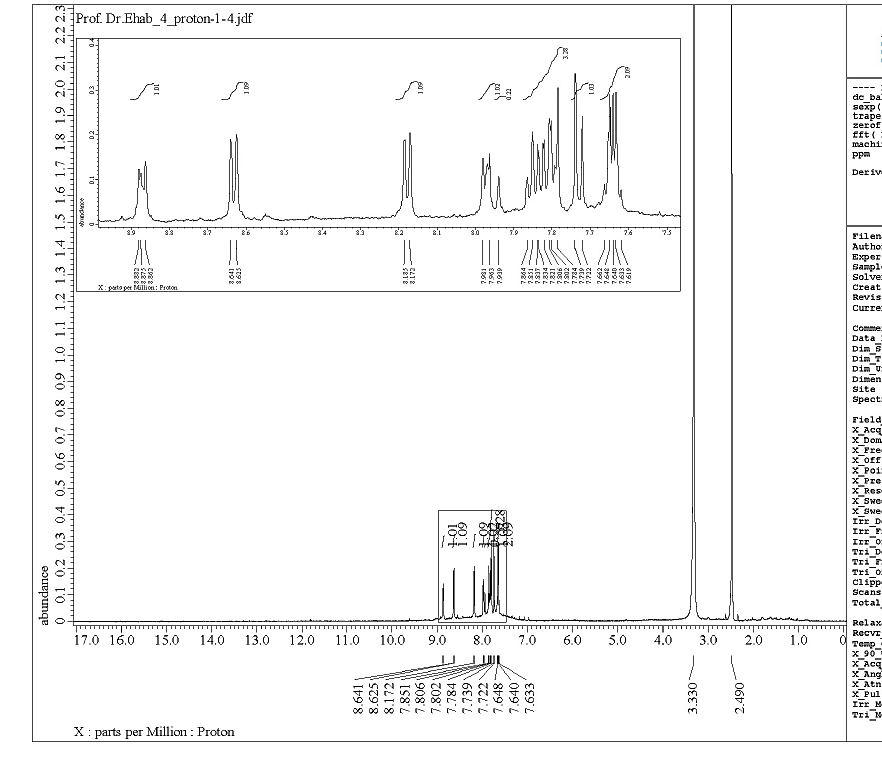
**

**Figure (S45): ^1^H NMR spectrum of compound 16.**

**
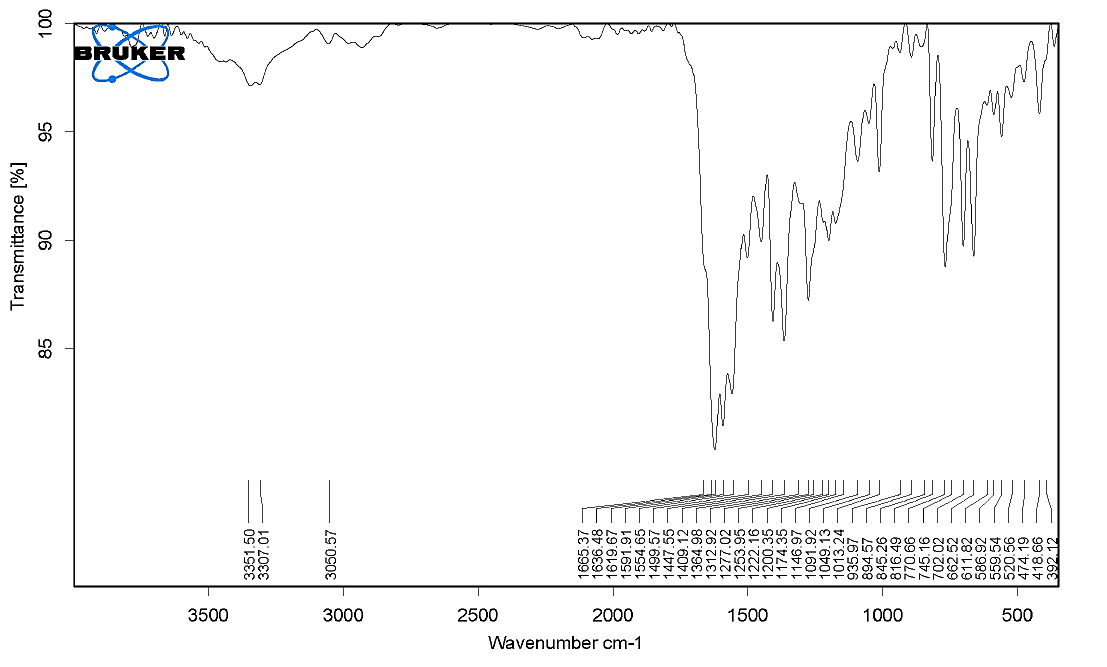
**

**Figure (S46): IR spectrum of compound 17.**

**
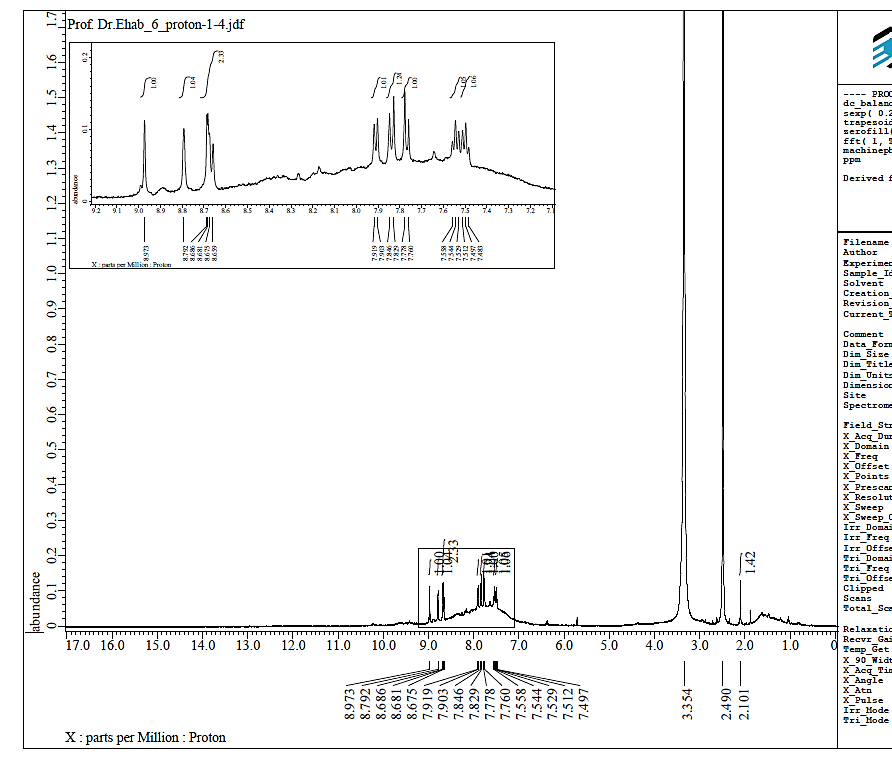
**

**Figure (S47): ^1^H NMR spectrum of compound 17.**

**
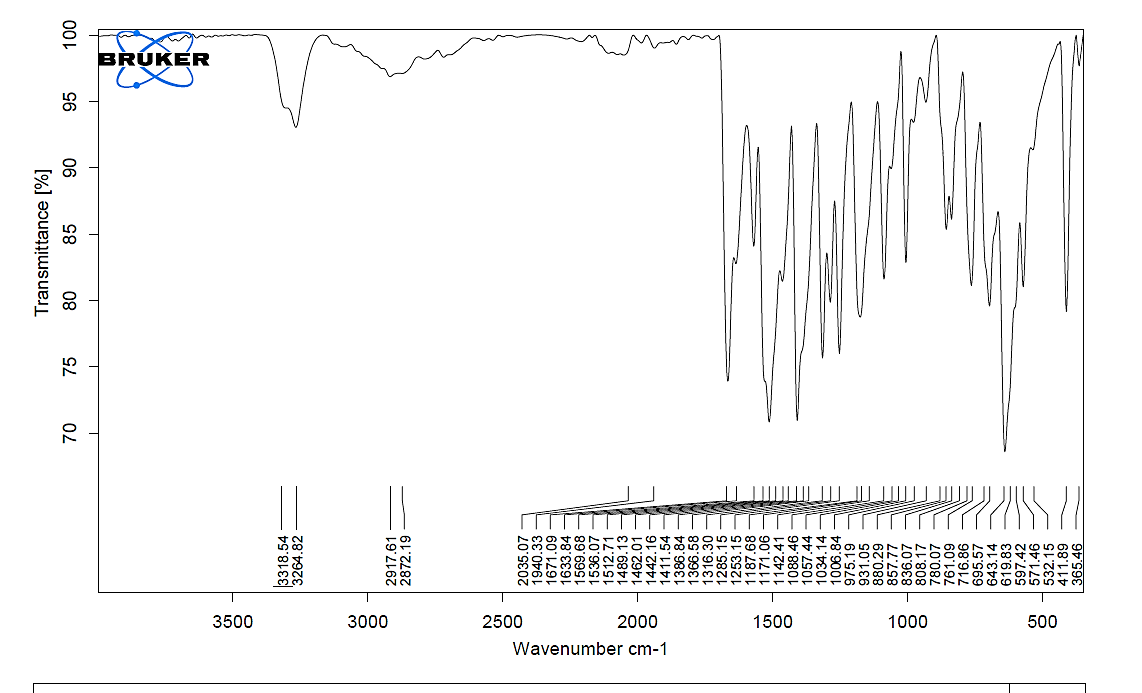
**

**Figure (S48): IR spectrum of compound 19a.**

**
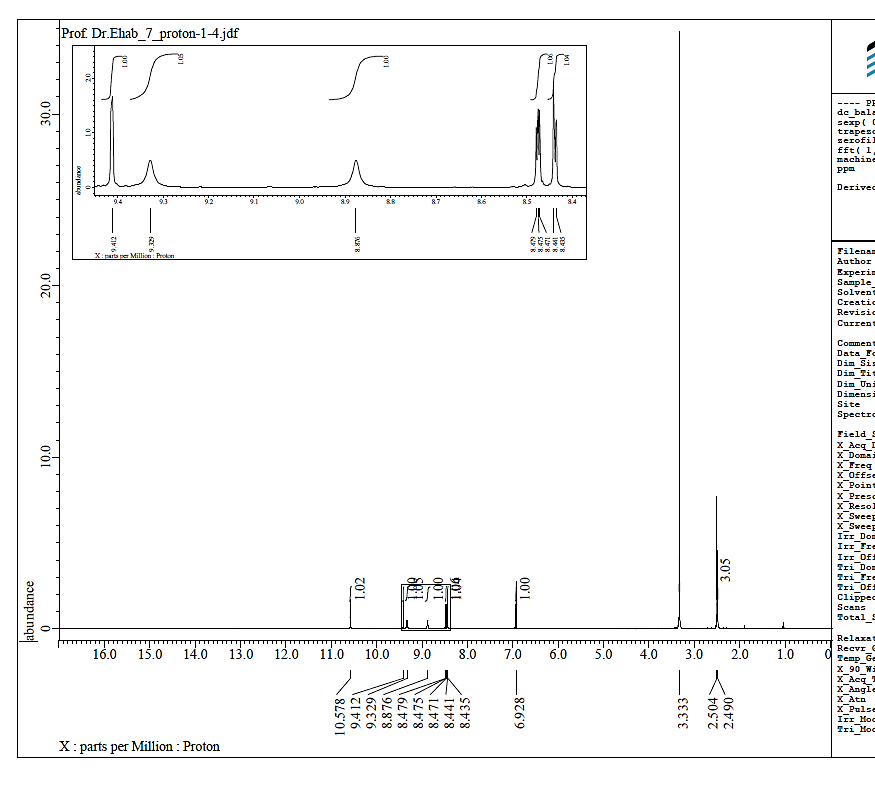
**

**Figure (S49): ^1^H NMR spectrum of compound 19a.**

**
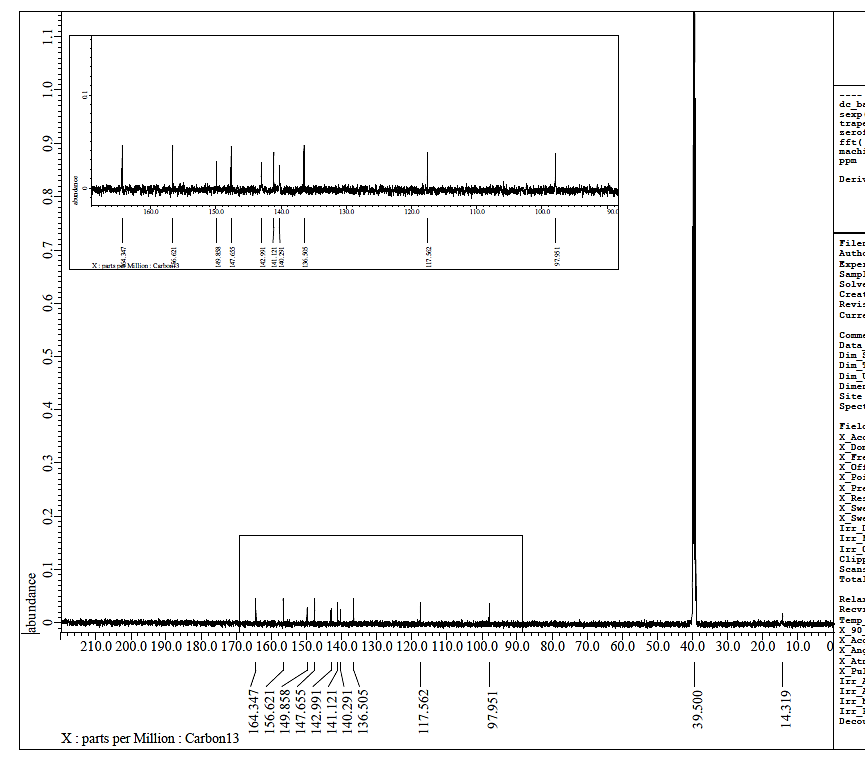
**

**Figure (S50): ^13^C NMR spectrum of compound 19a.**

**
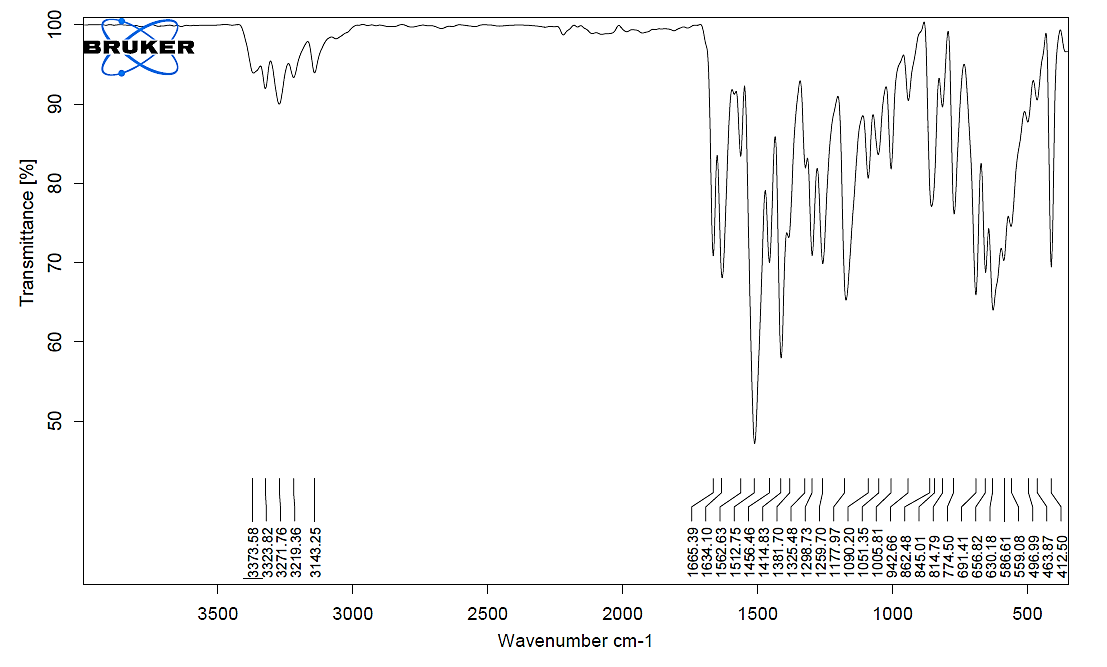
**

**Figure (S51): IR spectrum of compound 19b.**

**
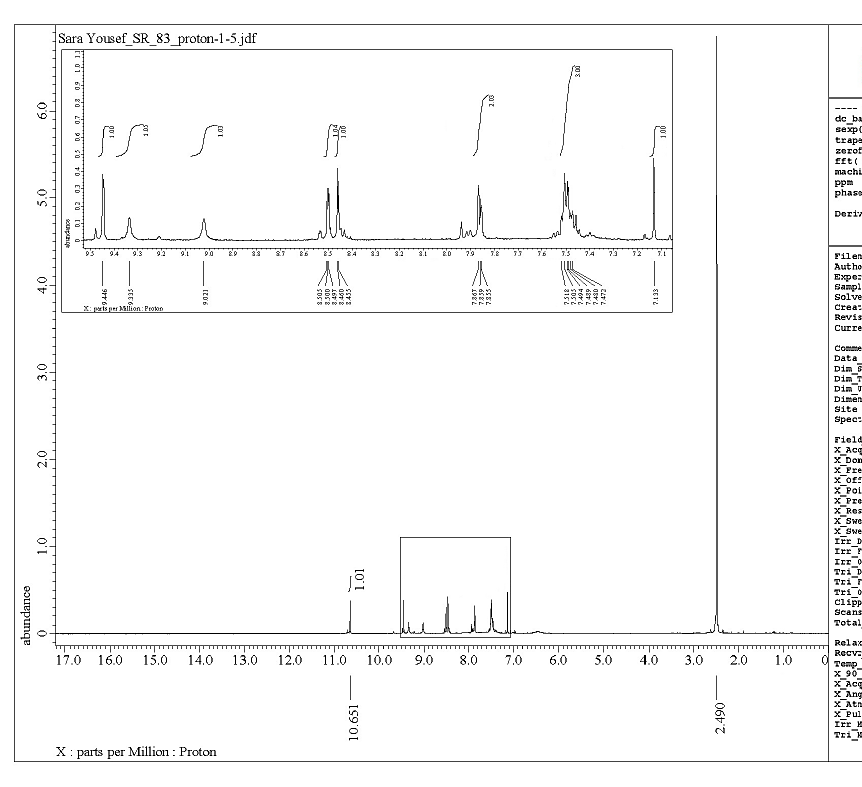
**

**Figure (S52): ^1^H NMR spectrum of compound 19b.**
